# Linking the Belowground Microbial Composition, Diversity and Activity to Soilborne Disease Suppression and Growth Promotion of Tomato Amended with Biochar

**DOI:** 10.1038/srep44382

**Published:** 2017-03-13

**Authors:** Amit K. Jaiswal, Yigal Elad, Indira Paudel, Ellen R. Graber, Eddie Cytryn, Omer Frenkel

**Affiliations:** 1 Department of Plant Pathology and Weed Research, Institute of Plant Protection, Agricultural Research Organization (ARO), The Volcani Center, Bet Dagan 50250, Israel; 2 Department of Soil Chemistry, Plant Nutrition and Microbiology, Institute of Soil, Water and Environmental Sciences, The Volcani Center, Bet Dagan 50250, Israel; 3 Department of Plant Pathology and Microbiology, The Robert H. Smith Faculty of Agriculture, Food and Environment, The Hebrew University of Jerusalem, P. O. Box 12, Rehovot, 76100, Israel; 4 Department of Soil and Water Sciences, The Robert H. Smith Faculty of Agriculture, Food and Environment, The Hebrew University of Jerusalem, P. O. Box 12, Rehovot, 76100, Israel

## Abstract

Biochar, in addition to sequestering carbon, ameliorating soil, and improving plant performance, can impact foliar and soilborne plant diseases. Nevertheless, the mechanisms associated with suppression of soilborne diseases and improved plant performances are not well understood. This study is designed to establish the relationships between biochar-induced changes in rhizosphere microbial community structure, taxonomic and functional diversity, and activity with soilborne disease suppression and enhanced plant performance in a comprehensive fashion. Biochar suppressed Fusarium crown and root-rot of tomato and simultaneously improved tomato plant growth and physiological parameters. Furthermore, biochar reduced Fusarium root colonization and survival in soil, and increased the culturable counts of several biocontrol and plant growth promoting microorganisms. Illumina sequencing analyses of 16S rRNA gene revealed substantial differences in rhizosphere bacterial taxonomical composition between biochar-amended and non-amended treatments. Moreover, biochar amendment caused a significant increase in microbial taxonomic and functional diversity, microbial activities and an overall shift in carbon-source utilization. High microbial taxonomic and functional diversity and activity in the rhizosphere has been previously associated with suppression of diseases caused by soilborne pathogens and with plant growth promotion, and may collectively explain the significant reduction of disease and improvement in plant performance observed in the presence of biochar.

Loss of soil organic matter and associated loss of soil microbial diversity and activity due to intensive agriculture has contributed to an increase in soilborne plant diseases^1^. Soilborne diseases cause considerable agricultural crop losses every year, and management of soilborne diseases is identified as one of the top farm management issues faced by farmers around the world^2^. Agricultural chemicals are commonly used for management of soilborne diseases. However, the high frequency of chemical use, non-target effects, development of pathogen resistance to chemical pesticides, risks to human health and the surrounding environment, and phasing out of some effective soil fumigants like methyl bromide have encouraged the development of alternative methods for disease management^3^. Recently, the application of biochar (the solid co-product of biomass pyrolysis) to soil has been shown to have the potential to suppress plant diseases caused by both soilborne and foliar pathogens^4^,^5^,^6^,^7^, which may be a function of biochar dose, feedstock, and production conditions^7^. This adds to the list of benefits that biochar use as a soil amendment may bring, which include carbon sequestration, soil amelioration and improvement in crop yield^6^,^8^,^9^. In the literature to date, biochar has been reported to affect the progress of foliar diseases in 6 pathosystems (*i.e*. plant/pathogen system) caused by 4 foliar pathogens^10^,^11^. Since the biochar in these studies was added to the soil, and the disease-causing pathogen was foliar, it could be inferred that the biochar played a role in mediating systemic plant responses to the foliar diseases^4^. It was later shown that biochar in the growing medium could mediate up-regulation of defense-related genes in plants^12^. Biochar has been additionally reported to affect the progress of soilborne diseases in 22 pathosystems caused by 11 soilborne pathogens^10^,^11^. By and large, mechanisms responsible for attenuating severity of diseases caused by pathogens in the soil were not fully addressed. As a crucial step in establishing a prudent usage of biochar, the mechanisms involved in disease suppression and growth promotion need to be further understood. Soilborne disease progress may be affected by biochar due to direct effects on the factors occupying the three vertices of the ‘disease triangle’ (environment, host plant, pathogen), as well as indirectly via its influence on the rhizosphere microbiome and its subsequent effect on those three major factors^7^,^13^. Mechanisms possibly linked to the impact of biochar on progress of diseases caused by soilborne pathogens include: (i) improved nutrient supply and availability; (ii) enhanced soil physiochemical characteristics; (iii) altered pathogen growth, survival, and activity; (iv) induced systematic plant defense mechanisms; and (v) altered soil microbial abundance, diversities, and activities.

The rhizosphere microbiome has been linked to plant growth promotion and soilborne pathogen suppression in many ways: increasing nutrient availability and uptake, producing plant growth stimulating hormones, competing with pathogens for resources, producing compounds that inhibit pathogens, parasitizing pathogens, or even inducing systemic plant resistance against diseases and pests in many plant species^14^,^15^,^16^. In addition, high microbial taxon and functional diversity and activity previously are associated with plant growth promotion, plant defense systems activation and soilborne disease suppression^17^,^18^,^19^,^20^,^21^. Rhizosphere microbiome composition, diversity, and activity can be altered by plant species, plant developmental stage, and various environmental factors, including soil type, pH, moisture, temperature^22^,^23^, and the addition of soil organic amendments^17^,^19^. To date, studies of the influence of biochar on soil microbial community structure and promotion of beneficial microorganisms have yielded rather contradictory results. Some reported that biochar amendment significantly altered microbial community composition, while others observed only minor changes^24^,^25^. However, most of the studies targeted mainly bulk soils; only two targeted rhizosphere microbiomes and their impact on plant disease^26^,^27^. Both those studies tested the influence of biochar on diseases caused by foliar pathogens, which are different from soilborne pathogens, in that foliar pathogens do not interact directly with the biochar, soil, plant roots, and the rhizosphere microbiome.

To date, no comprehensive relationship has been established between biochar-induced changes in microbial community structure, diversity and activity, and the concomitant suppression of diseases caused by soilborne pathogens and enhanced plant performance. Our research objective was to examine this relationship. The model pathosystem was tomato (*Solanum lycopersicum*) and *Fusarium oxysporum* f. sp. *radicis lycopersici* Jarvis and Shoemaker (FORL). This soilborne Ascomycete, the causal agent of Fusarium crown and root rot (FCRR), is a destructive pathogen of tomato in both greenhouse and field production, reducing yields by 15–65%^28^.

## Materials and Methods

### Biochar and plant growing medium

Two contrasting types of biochar, EUC-600, produced from eucalyptus wood chips at 600 °C pyrolysis temperature (highest treatment temperature; HTT) and GHW-350, from greenhouse pepper plant wastes produced at 350 °C HTT, were used throughout the present research. These two feedstocks differ substantially in structure and chemistry from each other (e.g., ligneous versus cellulosic, respectively). Both were prepared in an in-house, semi-continuous, batch pyrolysis reactor operated in direct retort mode at the given HTT (BEK, All Power Labs, San Francisco, California). Biochars were ground into a powder of <0.5 mm particles and stored in sealed containers. The most pertinent characteristics of biochars are detailed in Supplementary Tables S1 and S2, with more details available in previous publications^6^,^7^. The commercial potting mix was a horticultural grade peat:tuff 7:3 vol:vol mixture (Shaham Givat-Ada, Israel). Pertinent characteristics and the base-level microbiome (bacterial order level) of the potting mixture are detailed in Supplementary Tables S1 and S3.

### Plants and growing conditions

Tomato seeds (*S. lycopersicum*, cv. M-82, Zeraim Gedera, Israel) were surface sterilized with 1.5% NaOCl and rinsed three times with sterile water. Seeds were sown in a tray containing the potting mixture that was previously homogenized with or without EUC-600 and GHW-350 biochar (0, 0.5, 1, and 3% wt:wt). After germination, a single tomato seedling (21-day-old) was transplanted to each pot (0.5 L, diameter = 10 cm) containing potting mixture with or without biochar (0–3% wt:wt), that was either pathogen-free or inoculated with FORL as described below. Each treatment included four replicates (*i.e*. four blocks) with five plant per replicate (total 20 plants per treatment). Transplanted seedlings were maintained in a greenhouse at 22 ± 1 °C, and were fertilized and irrigated with drippers two times per day with 5:3:8 NPK fertilizer (irrigation water was planned to have total N, P, and K concentrations of 120, 30, and 150 mg liter^−1^, respectively; EC 2.2 dS m^−1^), allowing for 25–50% drainage. By growing the seedlings in well-structured soilless media and providing optimal fertigation, differences in nutritional or water status between the treatments at all levels of biochar amendment (0, 0.5, 1, and 3%) were avoided.

### Pathogen growth, inoculation, and disease evaluation

FORL (isolate 8112, kindly provided by Prof. Jaacov Katan, The Hebrew University) was cultured on potato dextrose agar (Difco Laboratories, Detroit) in Petri dishes and incubated at 25 °C for 5 days. Five FORL agar disks (7 mm) were placed in 250 ml Erlenmeyer flask containing 80 ml pearl millet seed (*Pennisetum glaucum*) that was previously soaked in water overnight and then autoclaved for two consecutive days, 24 hours apart. The FORL culture was incubated for 12 days at 25 °C. After 12 days, FORL-infested millet seeds were mixed with the potting mixture at a concentration of 0.75% (wt:wt). Non-infected millet seeds which underwent the same autoclave preparation served as the non-inoculated control treatment.

Five plants of each replicates were used to calculate the percentage of the collapsed plants per replicate and was recorded daily until there was no more disease progression (17 days after transplanting, DAT). Disease severity (percentage of the crown and root rot) was assessed visually 24 DAT by examining the plant roots.

### Tomato growth parameters

Tomato growth parameters were evaluated at the end of the experiments (24 DAT). Plant height was measured from stem base to top. Total number of leaves of plant and fresh weight of root were measured. Plant shoots were dried in an air circulation 60 °C oven for 7–10 days until dry weight was unchanged.

### Plant shoots nutrient sampling and measurements

Plant shoots were sampled at the end of experiments. Shoots were washed with distilled water and then, dried for 1 week in a ventilated oven at 60 °C. The dry tissue was ground and sieved through a 20-mesh sieve. One hundred-mg samples were wet ashed with H_2_SO_4_- H_2_O_2_ and analyzed for N, P and K. Ashing in HClO_4_-HNO_3_ was used to analyze Ca, Mg, Zn, and Mn. Element concentrations were determined as follows: total N and P by autoanalyzer (Zellweger Analytics, Milwaukee, WI); K by flame photometer (M410, Sherwood Sci. Ltd); Ca, Mg, Zn, and Mn by atomic absorption spectrophotometer (AAnalyst 400, Perkin Elmer).

### Plant physiology parameters

For photosynthetic pigment analysis, five leaf discs (1 cm diameter) from each replicate were sampled at end of experiment. Leaf samples were inserted into a glass bottle containing 5 ml of 100% methanol and were extracted for 24 hours in the dark; determination of total chlorophylls and carotenoids were conducted according to Lichtenthaler^29^.

For membrane leakage analysis, roots, hypocotyls and leaves of tomato plants were excised and were rapidly rinsed with double distilled water (DDW) upon collection, carefully placed into prepared vials with DDW, and shaken for 24 hours after which time electrical conductivity (EC) was measured by EC meter. The samples were then autoclaved (121 °C for 20 min), and after cooling to room temperature, EC was determined again. Membrane leakage was calculated as the ratio of the EC at 24 hours (before autoclave) to EC following autoclave.

Net photosynthesis rate (A_n_), stomatal conductance (G_s_), transpiration rate (T_r_), and electron transport rate (ETR) were measured from 9:00 to 11:00 h with a portable photosynthesis system (Li-6400XT, LI-COR Inc., Lincoln, NE) at leaf temperature 30 °C, light 1200 μmol m^−2^ s^−1^, 10% blue light, 400 μmol CO_2_ mol^−1^.

### Growing medium moisture content and pH

Plant growing media was sampled from each treatment with three replicates to measure potting mixture moisture content (%) and pH (aqueous extracts of potting mixture and double distilled water, 1:20 w:v, shaken for 24 h) by gravimetric method and pH meter, respectively.

### Root colonization and survival of FORL in potting mixture

Quantification of *Fusarium* spp. in the roots of inoculated tomato transplants was conducted by root maceration method. Tomato roots were collected 1 and 3 days after inoculation (before symptom appearance, *i.e*. asymptomatic plants) and 5 and 8 days after inoculation (when symptoms generally appeared, *i.e*. symptomatic plants). The roots were cut and thoroughly washed in 30 ml sterile saline water (0.85% NaCl, w:v) by stirring for one min, three sequential times. The washed roots were blotted, weighted, and macerated with 10 ml sterile saline water; the paste was plated on a modified peptone PCNB (Pentachloronitrobenzene) medium^30^ using the plating dilution technique, and incubated for 5 days at 25 °C. Results were expressed as colony forming units (CFU) g^−1^ fresh root weight and data were transformed to log CFU values.

*Fusarium* inoculum was mixed with the potting mixture with or without biochars to assess the effect of biochar on survival of *Fusarium* in potting mixture. Assessment of *Fusarium* populations was carried out using the serial dilution method on a modified peptone PCNB medium throughout 25 days. Results were expressed as CFU g^−1^ dry sample. CFU g^−1^ dry potting mixture data were transformed to log CFU values and the FORL survival curve was drawn. The slope of the logit-transformed survival curve over time was used to estimate the apparent reduction rate.

### *In-vitro* direct toxicity of biochar to FORL

Direct toxicity of original unwashed biochar, washed biochar, and aqueous extract of biochar amended in Czapek dox agar medium (Difco Laboratories, Detroit) at varying concentrations (0, 0.5, 0.75, 1, and 3%, w-v) was studied using an *in-vitro* contact assay to evaluate radial hyphal growth inhibition as previously described by Jaiswal *et al*.^7^. Washed biochar and aqueous extract of biochar was prepared as follows: biochar at varying concentrations (0–3%, w:v) in deionized water was shaken at 150 RMP for 24 hours at 25 °C and then allowed to settle. After settling, the solution and solid phase were separated by centrifuge (10000× g, 10 min, and 25 °C). The pellet and supernatant were used as washed biochar and aqueous extract of biochar, respectively. pH values of the amended media were adjusted according to the non-amended control media (pH 7.3 ± 0.1).

### Abundance and identification of culturable microorganisms

Plants were removed from pots and roots were manually shaken. Potting mixture attached to the tomato root surfaces (rhizosphere) amended with either 0% (non-amended control) or 1 and 3% EUC-600 and GHW-350 biochar were sampled to quantify six types of culturable microorganisms by serial dilution plating on selective media. General bacterial populations were quantified on nutrient agar supplemented with 1 ppm benomyl^31^, filamentous fungi, *Trichoderma* spp. and yeast on PDA medium (Difco Laboratories, Detroit) supplemented with 250 ppm chloramphenicol (Sigma Aldrich, Israel) and 50 ppm Rose Bengal stain^32^. *Actinomycetes* spp. on were evaluated on KSTR medium^33^ and Fluorescent *Pseudomonas* spp. were evaluated on Kings B medium^34^. Microbe colonies were counted to calculate CFUs g^−1^ dry potting mixture. Taxonomic identification of specific *Pseudomonas* strains was conducted by using 16S rRNA gene sequencing with the general bacterial primer pair 11F and 1392R^35^. After elimination of chimeric sequences using DECIPHER-Find Chimeras (http://decipher.cee.wisc.edu/FindChimeras.html), partial 16S rRNA gene sequences were compared to sequences from the NCBI and RDP databases using Blast and the RDP classifier (https://blast.ncbi.nlm.nih.gov/Blast.cgi and https://rdp.cme.msu.edu/classifier/classifier.jsp, respectively). Sequences were deposited into the NCBI under Genbank accession numbers KY172831 and KY235785-KY235789.

### Potting mixture sample collection and DNA extraction

Potting mixture attached to root surfaces was sampled as described above from the rhizosphere of tomatoes amended with either 0% (non-amended control) or 3% GHW-350 biochar (as 3% amendments provided maximum disease control against FORL and plant growth enhancement) before the FORL inoculation. Each treatment consisted of four biological replicates for DNA extraction and each replicate contained combined potting mixture samples from five individual plants in order to reduce variability. Samples were stored at −80 °C until DNA was extracted. DNA was extracted from 0.3 g of rhizosphere potting mixture with two technical repeats using a commercial soil DNA extraction kit (Exgene Soil SV, Geneall Biotechnology Co. Ltd., Republic of Korea) according to the manufacture’s protocol, including two cycles of bead beating for 45 s with 4.5 speed using FastPrep FP120 (Bio 101, Savant Instruments Inc., Holbrook, NY, USA). Extracted DNA yield and purity was measured by Nanodrop (ND-1000 Spectrophotometer, Wilmington, USA) and integrity by running in 1.5% agarose gel electrophoresis. Finally, extracted DNA was diluted to 10 ng μL^−1^ prior to PCR amplification.

### Illumina high-throughput sequencing of 16S rRNA gene amplicons

The DNA samples were PCR-amplified using the CS1_515F (ACACTGACGACATGGTTCTACAG TGCCAGCMGCCGCGGTAA) and CS2_806R (TACGGTAGCAGAGACTTGGTCTGGACTACHVGGGTWTCTAAT) primer set^36^, which amplifies the V4 region of the 16S rRNA gene and contains adaptors for library preparation for next generation sequencing. PCR reactions (final volume of 50 μL) contained the following components: 25 μL Dream Taq PCR master mix (2x) (Thermo Scientific), 1.5 μL (10 μg μL^−1^) bovine serum albumin (Thermo Scientific), 2.5 μL (10 μM) of each primer and 3 μL (10 ng μL^−1^) DNA template. The PCR program consisted of an initial denaturation step of 94 °C for 180 s followed by 28 cycles of denaturation at 94 °C for 45 s, annealing at 50 °C for 60 s and elongation at 72 °C for 60 s. Cycling was completed with a final elongation step of 72 °C for 10 min. Amplification of a non-template control was performed in order to detect contamination during the preparations. The samples were stored at −20 °C until sent for sequencing. The sequencing stages were performed using Illumina MiSeq technology at the Research Resources Center of the University of Illinois at Chicago, according to the amplicon sequencing protocol of the DNA service facility (http://www.rrc.uic.edu/dnas). The sequence data generated in this study was submitted to the NCBI under bioproject number PRJNA354172.

### Sequence processing and bioinformatics data analysis

Pair-end FASTQ files were merged using PEAR. CLC software was used for quality and length trimming (retaining those longer than 225 bp with minimum quality score of Q30). The rest of the analysis was performed using Quantitative Insights into Microbial Ecology (QIIME; version1.9.1) pipeline^37^, unless stated otherwise. Sequences were grouped into operational taxonomic units (OTUs; 97% similarity cutoff) using UCLUST^38^. A representative sequence from each OTU was picked and aligned using PyNAST to the Silva 16S rRNA bacterial database (https://www.arb-silva.de/). Each representative sequence was assigned a taxonomy using the UCLUST algorithm and the Silva database. Utilizing the taxonomic assignments and the alignment of the representative sequences, an OTU table was created. Finally, sequences identified as chimeras by ChimeraSlayer, singletons, chloroplasts, and mitochondria were removed from the analysis. OTUs generated from the data processing were used to determinate β (between samples) and α (within samples) diversity. Taxonomic-based alpha diversity was calculated based on the total number of phylotypes (richness) and on Shannon’s diversity index (H′).

Bray–Curtis distances matrix, using most abundant OTUs (at least 5 sequences in total), was used to estimate the β-diversity, which was then visualized using nonmetric multidimensional scaling (NMDS) using the Paleontological Statistics Software Package (PAST; http://folk.uio.no/ohammer/past/). The heatmap was created using most abundant OTUs (at least 50 sequences in total) in QIIME. Statistically significant differences in bacteria taxonomic abundances and alpha diversity parameters as a result of biochar amendments was determined by Tukey-Kramer (HSD) test (α = 0.05) using JMP 12 software (SAS Institute, Cary, NC). Statistical differences between groups of samples were tested by permutational multivariate analysis of variance (PERMANOVA) and analysis of similarities (ANOSIM) available through PAST.

### Bacterial community-level physiological profiling

The community-level physiological profiles (CLPP) technique using Biolog GN plates (96-well plates containing 95 different carbon sources; Biolog Inc, Hayward, CA) was applied to elucidate the effect of biochar on the functional potential of rhizosphere microbial communities. A 2.5 g aliquot of potting mixture collected from the rhizosphere of tomatoes amended with either 0% (non-amended control) or 3% GHW-350 biochar was suspended in 22.5 mL of sterile 0.85% NaCl solution and incubated in a shaker for 1 hr at 25 °C. After settling for 10 min, the potting mixture suspensions were diluted 1:1000 and added (150 μL) to each of the 96 wells in the micro-plate and incubated at 25 °C in the dark. Color development in the wells was measured at 595 nm with an iMark microplate reader (BioRad, Richmond, CA) at 12 hours intervals for 120 h incubation period. Average well color development (AWCD) was determined for each plate according to the method of Garland^39^.

Non-metric multidimensional scaling (NMDS) ordination was used to test for potential differences in CLPP after normalizing the data by dividing the difference in absorbance for each substrate and the control well by the AWCD of the plate at 72 h^40^. In order to determine maximal utilization rates, the Biolog plates substrates were divided into seven functional groups (carbohydrates, carboxylic acids, polymers, amino acids, amines/amides, aromatic compound, phosphorylated chemicals and miscellaneous) as previously described^40^ and then maximum utilization rates were calculated for each functional group. The metabolic functional diversity of rhizosphere microbial community was descripted by the Shannon diversity index H′ based on the absorbance reading at 72 h according to Garland^39^. Richness (R) values were calculated as the number of oxidized C substrates, using an OD of 0.25 as the threshold for positive response^39^. Statistically-significant differences in AWCD, utilization rates of specific carbon groups, Shannon’s diversity index and richness as result of biochar amendments was determined by Tukey-Kramer (HSD) test (α = 0.05).

### Soil enzymatic microbial activity

Respiration rates of potting mixture amended with either 0% (non-amended control) or 3% GHW-350 biochar were measured using an acid-titration technique^41^. Briefly, a sample of 40 g of potting mixture in a closed jar was incubated for 24 h at 30 °C with 2 mL of 1 N NaOH trap, followed by acid titration to quantify the CO_2_ evolution. Oxidative activity was estimated by measuring dehydrogenase (DHA) activity^42^ using, 3, 5-triphenyltetrazolium chloride (TTC) as the substrate. The resulting product, 2 triphenyl formazan (TPF) was spectroscopically measured at 485 nm with a UV visible spectrophotometer (Genesys 10 UV scanning). Hydrolytic activity was estimated with the fluorescein di-acetate (FDA) method^43^. The amount of resulting product, fluorescein, was spectroscopically measured at 494 nm. The results of these measurements were expressed as production of CO_2_, TPF and fluorescein in mg per kg dry potting mixture for respiration, DEH and FDA assay, respectively.

### Experimental design and statistical analysis

All the plant experiments were conducted twice and the two experimental repeats were pooled and analyzed jointly with ‘experiment’ as an additional factor. Data were analyzed by ANOVA (analysis of variance) using JMP 12 software (SAS Institute, Cary, NC). To enable analysis of variance, percentage values were normalized by the arcsine square-root transformation^6^. Multiple comparisons of the means were conducted by using Tukey-Kramer (HSD) test (α = 0.05).

## Results

### Effect of biochar on tomato crown and root rot caused by FORL

**Figure 1:**
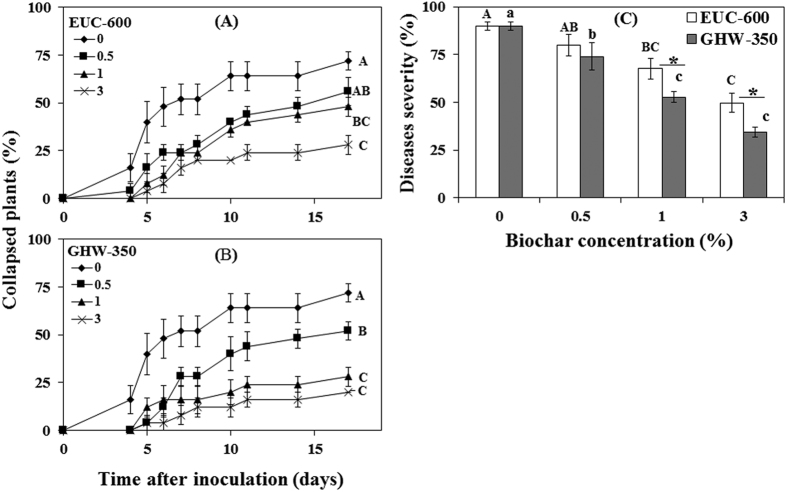
Effect of eucalyptus (EUC-600) (**A**) and greenhouse waste (GHW-350) (**B**) biochar amendments at concentrations of 0, 0.5, 1, and 3% on tomato plant collapse and final FORL disease severity of tomato plants (**C**). Columns labeled by a different capital letter and small letter are significantly different at *P* ≤ 0.05 according to Tukey Kramer HSD test within EUC-600 and GHW-350 biochars, respectively. Asterisk denotes the significant difference at *P* ≤ 0.05 according to Tukey Kramer HSD test between different biochar types at each concentration. Bars represent the standard error.

Plant collapse was observed in pots without biochar one day earlier than in pots amended with either EUC-600 or GHW-350 biochars (Fig. 1A,B). In the EUC-600 biochar treatments, the higher concentrations (1 and 3%) significantly reduced collapse by up to 61% as compared to the 0% biochar treatment (*P* = 0.0005; Fig. 1A). For GHW-350, all biochar concentrations (0.5, 1, and 3%) significantly reduced disease by up to 72% as compared to non-amended control (*P* < 0.0001; Fig. 1B). GHW-350 biochar treatments gave significantly higher disease reduction than EUC-600 biochar treatments (*P* < 0.05).

Disease severity was significantly reduced at 1 and 3% EUC-600 biochar by up to 44% as compared with the non-amended control (*P* = 0.0002; Fig. 1C). GHW-350 biochar significantly reduced disease severity at all concentrations compared with the non-amended control, by as much as 61% (*P* < 0.0001; Fig. 1C). At 1 and 3%, GHW-350 biochar gave significantly higher reduction of disease severity than EUC-600 biochar (*P* < 0.05; Fig. 1C).

The linear ANOVA model was applied to investigate the effect of biochar type and its interaction with concentration on FORL disease (Supplementary Table S4). All ANOVA models were highly significant. The factors “biochar types” and “concentration” had significant effects on all disease parameters. However, the interaction between these two main factors was insignificant.

### Effect of biochar on tomato plant growth parameters

**Figure 2:**
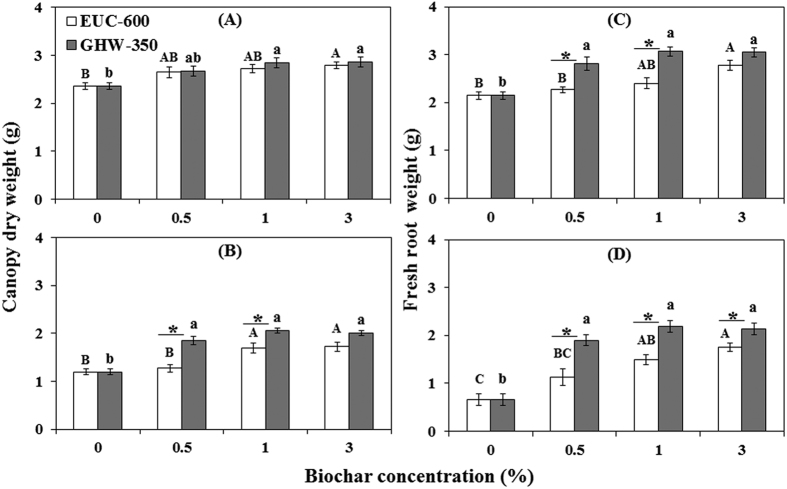
Effect of eucalyptus (EUC-600) and greenhouse waste (GHW-350) biochar amendments at concentrations of 0, 0.5, 1, and 3% on canopy dry weight (**A**,**B**); fresh root weight (**C**,**D**), without FORL inoculation (**A**,**C**) and with inoculation (**B**,**D**). Columns labeled by a different capital letter and small letter are significantly different at *P* ≤ 0.05 according to Tukey Kramer HSD test within EUC-600 and GHW-350 biochars, respectively. Asterisk denotes the significant difference at *P* ≤ 0.05 according to Tukey Kramer HSD test between biochar types at the same concentration. Bars represent the standard error.

Canopy dry weight and root fresh weight of tomato plants showed significant responses to both biochar treatments. In the absence of the pathogen, EUC-600 stimulated canopy and root growth with increasing concentration by as much as 18% (*P* = 0.0437) and 29% (*P* = 0.0068), respectively; GHW-350 biochar resulted in increased canopy and root weight by as much as 21% (*P* = 0.0174) and 42% (*P* = 0.0008), respectively, as compared to the non-amended control (Fig. 2A,C). In the presence of the pathogen, EUC-600 and GHW-350 biochars enhanced canopy weight by up to 43 and 71%, respectively (*P* < 0.0005) (Fig. 2A,B). Similarly, root weight was enhanced by EUC-600 and GHW-350 biochar by up to 164 and 229%, respectively, as compared with the control (*P* < 0.001) (Fig. 2C,D). Biochar had similar effects on plant height and leaf number per plant (Supplementary Table S6).

### Effect of biochar on tomato plant physiological parameters

**Figure 3:**
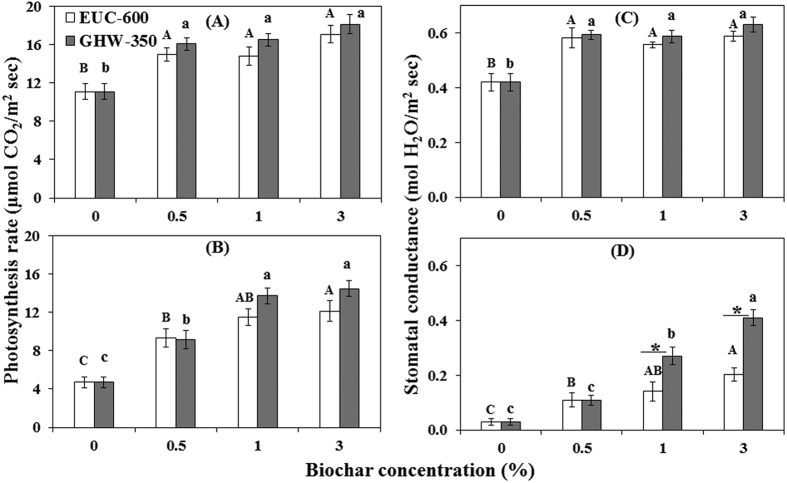
Effect of eucalyptus (EUC-600) and greenhouse waste (GHW-350) biochar amendments at concentrations of 0, 0.5, 1, and 3% on photosynthesis rate (**A,B**); stomatal conductance (**C,D**) without FORL inoculation (**A,C**) and with inoculation (**B,D**). Columns labeled by a different capital letter and small letter are significantly different at *P* ≤ 0.05 according to Tukey Kramer HSD test within EUC-600 and GHW-350 biochars, respectively. Asterisk denotes the significant difference at *P* ≤ 0.05 according to Tukey Kramer HSD test between biochar types at the same concentration. Bars represent the standard error.

The effect of EUC-600 and GHW-350 biochars on nine physiological parameters of the tomato plants is presented in Fig. 3A–D and in Supplementary Table S6. Net photosynthesis rate, stomatal conductance, transpiration rate and ETR were significantly higher under biochar amendments both in the presence and absence of the pathogen. In the EUC-600 treatments, the photosynthesis rate and stomatal conductance increased with increasing biochar concentration by up to 54% (*P* = 0.0018) and 40% (*P* = 0.001), respectively. In GHW-350 biochar treatments, the photosynthesis rate and stomatal conductance increased by up to 63% (*P* = 0.0001) and 50% (*P* = 0.0003), respectively, as compared to the non-amended control (Fig. 3A,C). In the presence of the pathogen, EUC-600 and GHW-350 biochars enhanced the photosynthesis rate by up to 157 and 207%, respectively (*P* < 0.0005; Fig. 3A,B). Stomatal conductance was enhanced by EUC-600 and GHW-350 biochars by up to 582 and 1270%, respectively, as compared with the non-amended control (*P* < 0.005; Fig. 3C,D). This general trend was also observed for transpiration rate and ETR (Supplementary Table S6). Photosynthetic pigment concentrations (total chlorophyll and carotenoid) and cell wall permeability of the tomato roots, hypocotyls and leaves as assessed by membrane ion leakage levels were not significantly different for plants grown in biochar-amended and non-amended potting mixture in absence of the pathogen (Supplementary Table S6). However, photosynthetic pigments in inoculated treatments were significantly higher in biochar treatments compared to non-amended control. The content of nutritional elements (N, P, K, Ca, Mg, Zn, and Mn) in the tomato shoots of the non-inoculated treatment were not significantly affected by either biochar amendments (Supplementary Table S7) and was within the optimal range for tomato plants^44^. Water holding capacity of growing media was also not influenced by biochar amendments but increased by 0.3 to 0.8 pH units, depending on the biochar concentration, from an initial of 6.5 to a maximum of 7.3 (Supplementary Table S7).

The linear ANOVA model was applied to investigate the effect of FORL inoculation, biochar type, concentration and their interaction on plant growth and physiological parameters (Supplementary Table S5). The main factors “FORL inoculation (I)”, “biochar types (B)” and “concentration (C)” had significant effect on all growth and physiological parameters. However, the two and three way interaction between different factors had no significant effect (except for stomatal conductance).

### *In vitro* direct toxicity effects of biochar on FORL

No significant radial mycelium growth inhibition was detected for either biochar in *in-vitro* assays as compared to the non-amended control (Supplementary Fig. S1), indicating that direct toxicity most likely cannot be regarded as a disease suppression mechanism in the tested pathosystem. No differences were found between unwashed and washed biochar.

### Effect of biochar on *Fusarium* root colonization and survival in potting mixture

The effects of EUC-600 and GHW-350 biochar at increasing concentrations (0, 1, and 3%) on *Fusarium* root colonization of tomato plant grown with or without biochar for 1 and 3 days after inoculation (before symptom appearance) and 5 and 8 days after inoculation (when symptoms generally appeared) are presented in Supplementary Fig. S2A. Biochar had no effect on *Fusarium* colonization after 1 day inoculation, but from 3 days onward, *Fusarium* colonization was significantly lower in treatments with EUC-600 and GHW-350 biochar (reduction in CFU by up to 71 and 85%, respectively, *P* < 0.005).

The effect of biochar on the survival of *Fusarium* in the potting mixture over the course of 25 days is shown in Supplementary Fig. S2B. While the *Fusarium* abundance after 5 days in both biochar treatments was statistically significantly lower than in the non-amended treatment (*P* < 0.001), the final concentrations of the *Fusarium* population in the biochar treatments were still relatively high (4.69 vs. 5.12 log CFU g^−1^ in EUC-600 and non-amended control treatments, respectively, and 4.60 vs. 5.12 log CFU g^−1^ in GHW-350 and non-amended control treatments, respectively).

### Effect of biochars on the abundance of culturable microbial populations

Both EUC-600 and GHW-350 biochar increased the counts of culturable general bacteria, fluorescent *Pseudomonas* spp., *Actinomycetes* spp., and *Trichoderma* spp. by 3-, 26-, 14-, and 2-fold, respectively as compared to the non-amended control (Table 1). Filamentous fungi and yeast counts were not significantly affected by either biochar amendments (Table 1). Several fluorescent *Pseudomonas* strains that were selected randomly from amongst those that were cultured and isolated from the biochar treatments showed antagonistic activity against FORL and inhibition of mycelium growth by up to 72%. Potential antagonistic strains identified using 16S rRNA gene sequencing showed highest similarity (*i.e*., 98–99%), to *Pseudomonas fluorescens, P. putida, P. koreensis, P. moraviensis*, and *P. monteilii*, all of which have been previously described as plant growth promoting or biocontrol agents^45^,^46^.

### Effect of biochar amendments on rhizosphere bacterial community composition and diversity

**Figure 4:**
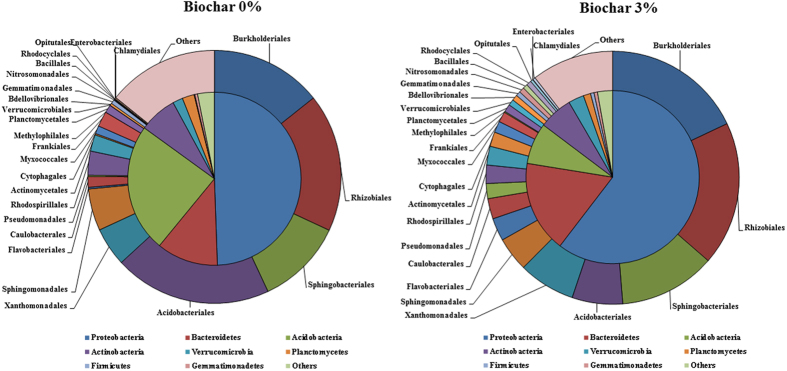
Effect of greenhouse waste (GHW-350) biochar on relative abundances of rhizosphere bacterial composition at Phylum level (inner circle) and Order level (outer circle) as identified by using the Illumina sequencing of 16S rRNA gene amplicons.

To explore the effect of biochar on bacterial community composition and diversity in the rhizosphere, Illumina sequencing of 16S rRNA gene amplicons was performed. Altogether, 8 samples (4 from non-amended control and 4 from 3% GHW-350 biochar amended potting mixture) were analyzed, generating a total of 400,734 reads with an average of 50,092 reads per sample from which 225,083 high quality sequences were selected for downstream analysis. Quality control screening and binning resulted in a total of 5038 unique OTUs. The vast majority of non-amended control (92.1%) and biochar-amended (91.6%) rhizosphere bacteria were associated with four primary phyla, *Proteobacteria, Bacteroidetes, Acidobacteria*, and *Actinobacteria*. The relative abundance of *Proteobacteria, Bacteroidetes, Verrucomicrobia*, and *Firmicutes* was significantly higher in the biochar-amended rhizosphere (*P* < 0.05), which represented 49.5 ± 0.42 vs. 60.4 ± 0.49%; 11.5 ± 0.64 vs. 17.1 ± 0.27%; 1.9 ± 0.05 vs. 2.9 ± 0.22%; and 0.1 ± 0.02 vs. 0.7 ± 0.07% of the total classified phyla in the non-amended and biochar-amended rhizosphere, respectively (Fig. 4). There were no differences in the abundances of either *Actinobacteria* or *Gemmatimonadetes*; these phyla constituted 7.1 ± 0.18 vs. 6.3 ± 0.68% and 0.5 ± 0.04 vs. 0.6 ± 0.06% for the non-amended and biochar-amended rhizosphere, respectively. In contrast, the relative abundances of *Acidobacteria* (24.1 ± 0.34 vs. 7.7 ± 0.11%; non-amended and biochar-amended rhizosphere, respectively) and *Planctomycetes* (2.2 ± 0.08 vs. 1.4 ± 0.04%; non-amended and biochar-amended rhizosphere, respectively) were significantly lower in the biochar-amended rhizosphere.

Relative abundances of major bacterial orders were significantly altered by biochar amendments, as illustrated in Fig. 4 and presented in Supplementary Table S8 with statistical analysis data. The most dominant bacterial orders that showed significantly higher abundances in the biochar treatments were *Burkholderiales, Xanthomonadales, Flavobacteriales, Caulobacterales, Pseudomonadales, Cytophagales, Myxococcales, Methylophilales, Verrucomicrobiales, Bdellovibrionales, Nitrosomonadales, Bacillales, Rhodocyclales, Opitutales*, and *Chlamydiales*. In contrast, the relative abundances of *Acidobacteriales, Rhodospirillales*, and *Frankiales* were substantially lower in the biochar-amended rhizosphere community.

A more comprehensive (genus level) assessment of the microbial communities in the biochar amended rhizosphere with specific focus on genera known for plant growth promotion, disease suppression and other possible ecological roles is shown in Table 2 (defined genera with abundance >0.1% were displayed). Biochar significantly stimulated the abundance of genera affiliated with soilborne disease suppression, plant growth promotion, and/or biological N_2_ fixation, most notably: *Massilia, Rhodanobacter, Sphingobium, Devosia, Rhizobium, Mesorhizobium, Brevundimonas*, and *Ochrobactrum* (by 2- to 10-fold); *Pseudomonas, Achromobacter, Stenotrophomonas, Comamonas, Ferritrophicum, Paenibacillus, Shinella*, and *Bacillus* (by 10- to 20-fold); *Microvirga* (by 91-fold) and *Flavobacterium* (by 203-fold); *Cellvibrio, Azospirillum*, and *Peredibacter* were detected only in the biochar treatment. In contrast, biochar significantly reduced other plant beneficial bacteria such as *Mucilaginibacter* (11-fold), *Sphingomonas* (3-fold) and *Burkholderia* (1.5-fold). In addition, biochar stimulated genera affiliated with organic compound degradation such as *Chthoniobacter* (3-fold), *Cytophaga* (6-fold), *Afipia* (9-fold) and genera affiliated to crude oil and aromatic compound degradation such as *Nitratireductor* (2-fold), *Sphingopyxis* (111-fold), and *Novosphingobium* (25-fold).

**Figure 5:**
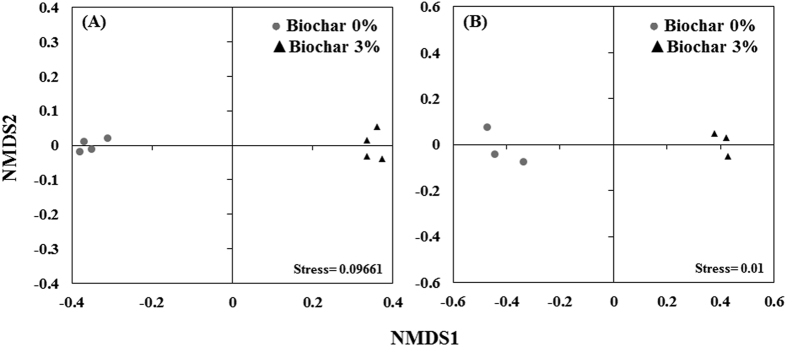
Beta diversity analysis to estimate the effect of greenhouse waste (GHW-350) biochar amendments on the rhizosphere microbial community composition and microbial metabolic potential. Taxonomic based NMDS ordination based on the Bray-Curtis similarity index, using most abundant OTUs (at least 5 sequences in total) (**A**) and Functional based NMDS ordination based on the Bray-Curtis similarity index of 95 carbon substrate utilization patterns obtained with Biolog Microplates (**B**).

**Figure 6:**
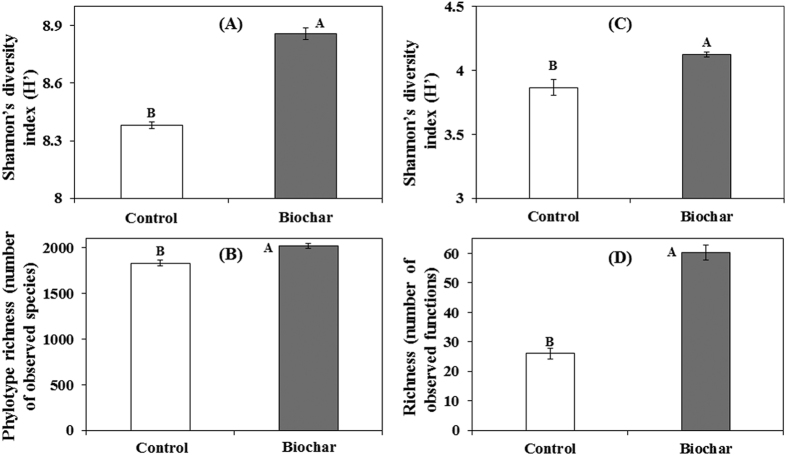
Effect of greenhouse waste (GHW-350) biochar amendments on alpha diversity of rhizosphere microbial community composition and microbial metabolic potential. Taxon based Shannon’s diversity index (**A**), Taxon based phylotype richness, using OTUs (97% identity) as identified by the Illumina sequencing of 16S rRNA gene amplicons using QIIME (**B**), Functional Shannon’s diversity index (**C**), Functional richness, based on 95 carbon substrate utilization patterns obtained with Biolog Microplates (**D**). Columns labeled by a different capital letter are significantly different at *P* ≤ 0.05 according to Student’s t-test. Bars represent the standard error.

Beta diversity analysis based on Bray-Curtis similarity matrix of the rhizosphere microbiome showed significant differences in bacterial community structure between the non-amended and biochar-amended rhizosphere (ANOSIM, *P* = 0.029, R = 1 and PERMANOVA, *P* = 0.0273). NMDS ordination based on Bray-Curtis distance matrix also showed substantial shifts in the rhizosphere bacterial community composition between biochar-amended and non-amended treatments (Fig. 5A). Furthermore, the heatmap of most abundant OTUs (at least 50 sequences in total) revealed high variability but distinct microbiome patterns between the two treatments (Supplementary Fig. S3). To gain further insight into the complexity of the rhizosphere bacterial communities, alpha diversity indices were estimated using Shannon’s diversity index (H’) and phylotype richness (R, number of observed OTUs), both of which demonstrated a higher bacterial diversity and richness in the rhizosphere amended with biochar than that in the non-amended control treatment (*P* < 0.005, Fig. 6A,B).

### Effect of biochar on microbial community metabolic potential, functional diversity and soil enzymatic microbial activity

**Figure 7:**
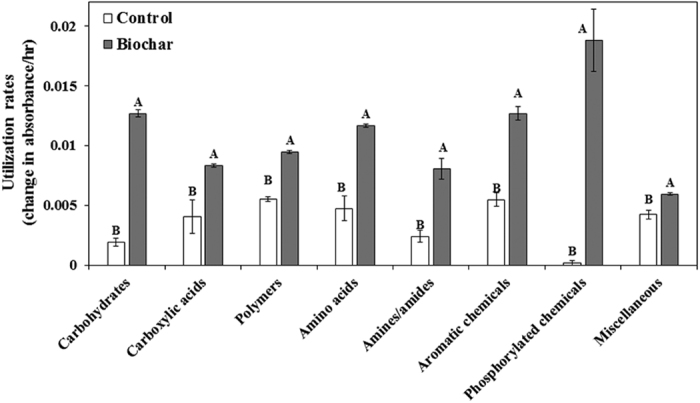
Effect of greenhouse waste (GHW-350) biochar amendments on rhizosphere microbial community carbon source utilization rates of specific carbon groups, based on 95 carbon substrate utilization patterns obtained with Biolog Microplates. Columns labeled by a different capital letter are significantly different at *P* ≤ 0.05 according to Student’s t-test. Bars represent the standard error.

Based on the carbon source utilization data, Bray-Curtis distance matrix based NMDS ordination showed substantial shifts in the microbial community metabolic potential of rhizosphere bacteria communities in the biochar amended and non-amended treatments (Fig. 5B). Generally, biochar amendments significantly increased the utilization rates for all of the Biolog plate substrate groups (*P* < 0.005, Fig. 7) by 72 to 9566%. Biochar amendments increased the functional Shannon’s diversity index (H’) and richness (R) by 7 and 132%, respectively, as compared to non-amended treatment (*P* < 0.005, Fig. 6C,D).

**Figure 8:**
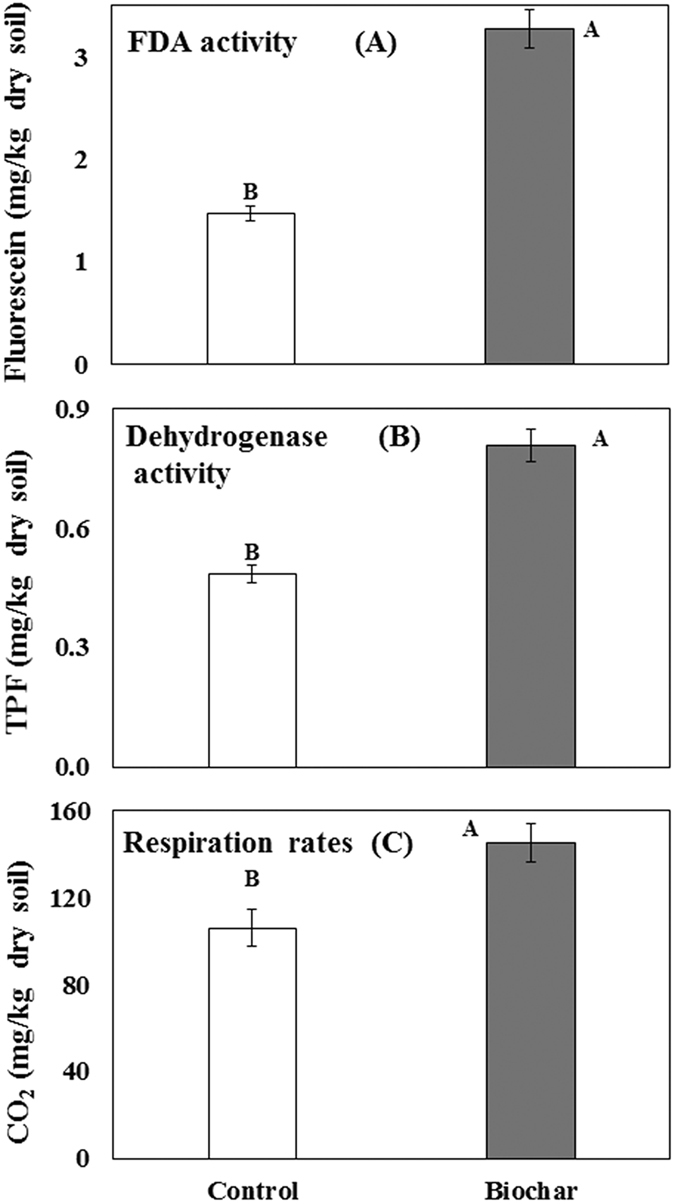
Effect of greenhouse waste (GHW-350) biochar amendments on microbial activities estimated by Fluorescein di-acetate activity (FDA; hydrolytic activity) (**A**), dehydrogenase activity (DHA, oxidative activity) (**B**), and respiration rates (CO_2_ emission) (**C**). Columns labeled by a different capital letter are significantly different at *P* ≤ 0.05 according to Student’s t-test. Bars represent the standard error.

Microbial activities estimated by dehydrogenase activity (oxidative activity), FDA activity (hydrolytic activity) and CO_2_ emission (respiration rates) were significantly increased following biochar amendments (*P* < 0.005), showing 122, 67, and 37% increase, respectively, as compared to the non-amended treatment (Fig. 8A–C).

## Discussion

Biochar added to a soilless potting mixture suppressed FORL crown and root rot of tomato and simultaneously improved tomato plant growth. By and large, biochar concentration and type had significant effects on plant performance and disease suppression, which increased with biochar concentration. There were no effects of biochar amendments on leaf nutrient status, plant tissue stability, photosynthetic pigmentation, or potting mixture water status. However, net photosynthesis rate, stomatal conductance, transpiration rate and electron transport rate increased following biochar amendments. Biochar amendments significantly shifted microbial community structure and functional potential of rhizosphere microbial community. Amendment with biochar also resulted in an increase in microbial taxon and functional diversity, microbial activities, and abundance of several groups closely related to biocontrol and plant growth promoting agents.

Previous studies are rather contradictory regarding the influence of biochar on the progress of soilborne diseases. Some studies observed an increase in disease severity^47^ as a result of biochar amendment; whereas others showed minor effects^48^ or no effects^49^ on disease suppression. Others reported a decrease in disease severity with either a U-shaped dose-response curve^6^,^7^,^50^ or linear dose-response curve^5^. Moreover, the mechanisms responsible for attenuating soilborne disease were not fully addressed. Infection of the tomato plant by FORL is mediated by synergistic activities of an array of cell wall degrading enzymes and phytotoxins^51^ that lead to plant cell wall disruption^28^. Thus, it could be suggested that higher plant cell wall rigor may be one potential explanation for enhanced plant resistance to FORL^52^. However, in our study, no differences in root or hypocotyl membrane leakage were observed between plants grown with and without biochar, indicating that the biochar had no significant influence on the cell wall and membrane traits. On the other hand, we observed increased tomato root growth in response to biochar amendments in both inoculated and non-inoculated treatments. As previously reported in the case of compost soil amendments, more vigorous root systems and formation of physical barriers at penetration sites are important for the ability of tomato plants to restrict FORL growth and development^53^. In theory, nutrients supplied by biochar^9^ or nutrients and moisture which are made more available by the presence of biochar^54^ could improve plant vigor and hence influence the ability of the pathogen to infect roots or growth in soil, but in this work, there was no impact of biochar amendments on either leaf nutrient or potting mixture water status, indicating that other mechanisms are probably involved.

In general, FORL-tomato interaction reduces photosynthesis efficiency, stomatal conductance, transpiration rate and other physiological activities in the plant, as a result of occlusion of conductive vessels with fungus mycelium or from toxicity of substances excreted by *Fusarium*^55^. Decrease in water movement suppress rates of photosynthesis, CO_2_ fixation, and transpiration, and also reduces the activity of electron-transport chain in chloroplasts and stomatal conductance^56^. In the present study, biochar improved the photosynthesis efficiency, stomatal conductance, transpiration rate and electron transport rate both in the absence and presence of the pathogen and even compensated for some deleterious effects of FORL on growth and the physiological condition of the tomato plant. This might be due to a decrease in FORL root colonization caused by biochar addition (Supplementary Fig. S2A) or possibly due to upregulation of certain protective hormonal pathways. Viger *et al*.^57^ found substantial stimulation of plant genes regulating plant growth hormones and photosynthetic machinery in *Arabidopsis* in the presence of biochar. Since the photosynthetic pigment content (chlorophyll and carotenoid) was not affected by biochar in the current study, the increase in the photosynthesis rate could possibly have been caused by an increase in the efficiency of the chloroplast, such as occurs as a result of changes in chloroplast structure or function^58^,^59^; this hypothesis remains to be tested.

Biochar was not directly toxic to the pathogen, as evidenced by the lack of biochar impact on radial mycelium growth, nevertheless, survival rates of the *Fusarium* were reduced in the biochar treatment both in the potting mixture and to an even greater extent in the vicinity of the tomato roots. Such a result was reported previously in other pathosystems^7^,^60^. This strengthens the hypothesis that mechanisms other than direct toxicity, such as an increase in beneficial microorganisms, microbial abundance, diversity and activity, induction of systemic resistance either by biochar-borne chemicals and/or biochar induced microbes may be involved in the observed disease suppression.

Adding biochar to the growing media introduced aromatic and organic compounds such as phenol, lactic acid, glycerol, hexanoic acid, butyric acids, benzoic acid^7^ (Supplementary Table S2). Such compounds potentially could have induced changes in microbial community composition in the bulk potting mix and rhizosphere that might be involved in suppressing the soilborne pathogen and enhancing the plant resistance. Alternatively, low concentrations of such chemical compounds could indirectly enhance plant growth and resistance^7^,^13^. Furthermore, the porous structure of the biochar, its high internal surface area and its ability to adsorb organic matter, nutrients, water and gases could serve as habitat for growth and refuge to microbes, which protect them from soil grazers and predators^61^,^62^. The biochar-elicited increase in potting mixture pH (6.5 vs.7.3 in the non-amended control and biochar-amended treatment, respectively) may also have played a role in promoting bacterial growth^63^. Some studies have shown that different soil amendments which increase soil pH also cause decreases in soilborne diseases caused by *Fusarium* species^64^,^65^.

Microorganisms in general, and specific strains belonging to the genera *Pseudomon*as, *Bacillus, Streptomyces*, and *Trichoderma* in particular, are known for their ability to improve plant growth, to suppress pathogens and to induce plant systemic resistance against diseases and pests in many plant species^14^,^32^,^46^. Such a synergy could have occurred in the current study, where culturable microbe abundances of most of the tested groups were found to be significantly higher in the rhizosphere of biochar-amended treatments. Indeed, several fluorescent *Pseudomonas* strains that showed antagonistic activity against FORL shared high sequence identity with *Pseudomonas* spp. that have been previously described as plant growth promoting or biocontrol agents^45^,^46^. Furthermore, higher abundance of general and specific bacteria in the biochar-amended rhizosphere might have influenced FORL by competing for space and resources (competitive exclusion) as previously suggested^14^,^16^.

Illumina sequencing of 16S rRNA gene analyses demonstrated higher relative abundances of *Proteobacteria, Bacteroidetes*, and *Firmicutes* phyla, and lower relative abundances of *Acidobacteria* and *Planctomycetes* in the rhizosphere of the biochar treatments, similar to trends documented in previous studies in biochar-amended bulk soils and rhizospheres^24^,^25^,^26^,^27^,^66^,^67^. Our study was conducted in a potting mixture consisting of peat soil and tuff, a typical growing mix that has a very important role in horticulture and plant nurseries. Such a mixture is a good model system that provides decent conditions for growing plant. While it is different from some of the soils used in other studies (such as loam, vertisol, and sand), the fact that several studies employing dissimilar plant, soil, and biochar types revealed similar selective recruitment of fast-growing copiotrophic phyla, suggests this is typical for the biochar-amended rhizosphere. Further study should focus on impacts of biochar under different soil types and sub-optimal growth conditions. *Acidobacteria*, for instance, generally prefer oligotrophic environments and lower pH soils^63^,^68^; therefore, it is possible that the reduction in *Acidobacteria* was a response to the biochar-related shift to a more neutral and copiotrophic environment. Yet, it should be noted that a number of subgroups of *Acidobacteria* do not decrease in the pH range relevant to this study^63^.

It is a challenge to determine bacterial function based on taxonomic groups, let alone attempting to determine correlations between the biochar-stimulated microbiome and ecological services. Genera may contain species having harmful, beneficial or no effect on plant performance and disease suppression (Table 2). Nonetheless, genera that are known to have a number of members which act as plant growth promoting agents, biocontrol agents and plant systemic resistance inducers such as *Pseudomonas, Devosia, Flavobacterium, Cellvibrio*, and *Bacillus* (Table 2) were stimulated by biochar amendments. Microbes from these genera often display a potential to produce or modulate phytohormones such as indol-3-acetic acid (IAA, auxin), cytokinins, and gibberellins^16^, which may suggest a linkage between microbe-elicited plant physiology and growth, promotion, and plant survival under biotic stress. Moreover, biochar also stimulated chitinolytic bacteria and cellulolytic bacteria that have capability to digest fungal and oomycetes cell walls. Oligo-chitin and -glucan fragments released from fungal and oomycetes cell walls during enzyme hydrolysis are reported to be active elicitors of plant defense responses^69^,^70^. A number of studies have established a strong link between diversity and richness of the soil microbiome and enhanced plant productivity^21^,^71^, suppression of soilborne pathogens^19^,^20^,^72^,^73^ and other ecological services^74^,^75^,^76^. In this study, we found that biochar amendments substantially increased bacterial taxon diversity and richness. This may partially explain the significant reduction of soilborne disease and increase in plant performance observed in the presence of biochar. Soils with near-neutral pH are known to support greater diversity and richness of bacterial populations than acidic soils^22^,^63^. Yet, while the biochar amendments in this experiment did increase pH by up to 0.8 pH units, from 6.5 to 7.3, this pH range was not shown to have an effect on taxon diversity and richness^22^. Alternatively, increased organic diversity due to release of various organic compounds from biochar (Supplementary Table S2) may result in increased bacterial diversity and richness, as different species use slightly different resources^74^.

In general, microbial communities with a high genetic (taxon) diversity and richness are usually correlated with higher functional diversity and have the capacity to utilize more diverse carbon sources^77^. This improves plant performance^18^ and confers protection against soilborne diseases^73^. We observed a clear increase in functional diversity and richness of rhizosphere microbiome and increase in utilization of different carbon source groups following biochar amendments. The observed increase in metabolic potential may be linked to increased abundance of rhizosphere bacterial taxa known to degrade biopolymers, carboxylic acids, aromatic compounds, phenols, and crude oil (Fig. 7, Table 2), suggesting that biochar-amendments may result in proliferation of microbial communities that can utilize biochar-associated organic compounds (Fig. 7, Supplementary Table S2). Some members of *Pseudomonas, Streptomyces*, and *Bacillus* spp., which was stimulated by biochar amendments are known as antibiotic producers. In general, antibiotic and volatile organic compound producers are often resistant to a multitude of antibiotics^78^,^79^ and toxic organic compounds^80^ (Table 2), indicating that biochar may suppress sensitive microbial communities and proliferate resistant ones. Another possibility is that biochar induced changes in root exudation, as reported for tomato by Akhter *et al*.^60^, and may have stimulated microbial communities associated with carbohydrate and amino acid metabolism (Fig. 7). It is estimated that up to 40% of the carbon photosynthetically-fixed by plants is exuded as rhizodeposits (*e.g*. exudates, border cells, mucilage) in the rhizosphere, which are a major driving force in the regulation of microbial diversity and activity^81^.

Microbial activities estimated by dehydrogenase activity (oxidative activity), FDA activity (hydrolytic activity) and CO_2_ emission (respiration rates) were significantly increased in the present study, concurrent with plant growth promotion and soilborne disease suppression following biochar amendments. The microbial activity of different organic amendments, determined by these latter means has been strongly correlated with suppression of different soilborne diseases^17^,^82^.

**Figure 9:**
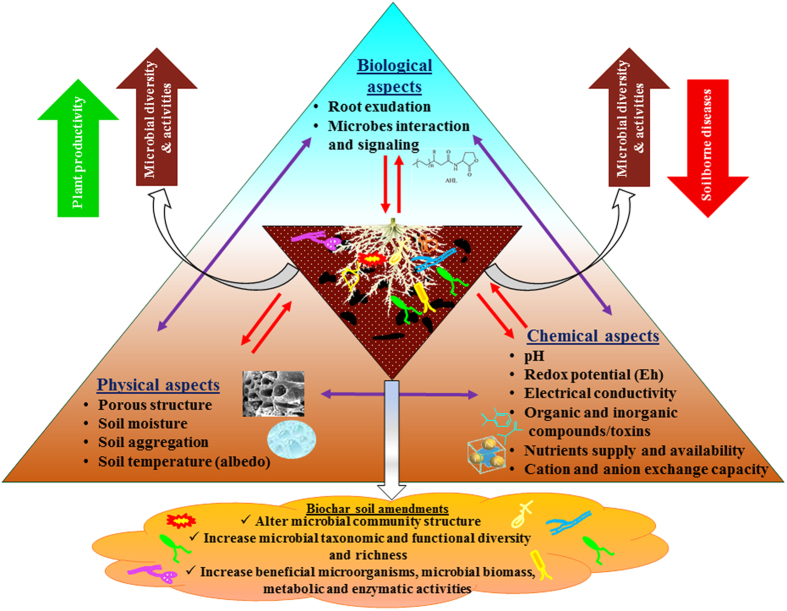
A rhizosphere microbiome centric view of the complex interactions between physical, chemical and biological components of the soil-biochar-plant-pathogen system. Biochar-induced changes in rhizosphere microbial structure, diversity and activity may be associated with direct or indirect effect of biochar on soil i) physical aspects (porous structure, soil moisture contents, aggregation and temperature); ii) chemical aspects (nutrients supply and availability, inorganic and organic compounds/toxins, pH, cation exchange capacity (CEC), electrical conductivity (EC), redox potential (Eh)); and iii) biological aspects (root morphology and exudations, interaction and signaling between microbes) of the rhizosphere. The increased microbial taxonomic and functional diversity and activity in the biochar amended rhizosphere is linked with suppression of soilborne diseases and with plant growth promotion.

In short, in this comprehensive study, we have shown there is a strong link between biochar-induced changes in microbial community structure, taxon-functional diversity and microbial activity and resultant soilborne disease suppression and enhanced plant performance. These results put the rhizosphere microbiome in the center of the broad, multi-mechanism model that envisions the impact of biochar on plant performance and health to be a function of complex interactions between many physical, chemical and biological components of the soil-plant-pathogen system^7^,^13^. This concept, whereby the rhizosphere microbiome plays a central role in the biochar effect, is illustrated in Fig. 9. It conforms to the emerging view that there is a strong link between taxon and functional diversity in the rhizosphere microbiome and enhanced ecosystem functioning^74^,^75^,^76^, plant productivity 21,71 and plant resistance to diseases caused by soilborne pathogens^19^,^20^,^72^,^73^.

## Additional Information

**How to cite this article:** Jaiswal, A. K. *et al*. Linking the Belowground Microbial Composition, Diversity and Activity to Soilborne Disease Suppression and Growth Promotion of Tomato Amended with Biochar. *Sci. Rep.* **7**, 44382; doi: 10.1038/srep44382 (2017).

**Publisher's note:** Springer Nature remains neutral with regard to jurisdictional claims in published maps and institutional affiliations.

## Supplementary Material

Supplementary Information

## Figures and Tables

**Table 1:** Effect of eucalyptus (EUC-600) and greenhouse waste (GHW-350) biochar at increasing concentration (0, 1, and 3%) on culturable rhizosphere microorganisms.

Microorganisms	Biochar type	Concentration (%)	CFU g^−1^ dry potting mixture (Average ± SE)	
General Bacteria	Control	0	6.77*10^7^ ± 6.85*10^6^	B# c
	EUC-600	1	9.43*10^7^ ± 6.92*10^6^	B
		3	1.54*10^8^ ± 4.99*10^6^	A
	GHW-350	1	1.41*10^8^ ± 4.96*10^6^	b
		3	1.88*10^8^ ± 6.64*10^6^	a
Fluorescent *Pseudomonas*	Control	0	3.22*10^4^ ± 9.90*10^3^	C c
	EUC-600	1	1.18*10^5^ ± 7.65*10^3^	B
		3	5.33*10^5^ ± 2.57*10^4^	A
	GHW-350	1	3.91*10^5^ ± 2.73*10^4^	b
		3	8.28*10^5^ ± 2.95*10^4^	a
*Actinomycetes* spp.	Control	0	1.04*10^5^ ± 1.76*10^4^	B c
	EUC-600	1	1.05*10^5^ ± 7.20*10^3^	B
		3	2.39*10^5^ ± 3.23*10^4^	A
	GHW-350	1	5.67*10^5^ ± 5.06*10^4^	b
		3	1.44*10^6^ ± 4.53*10^4^	a
Filamentous Fungi	Control	0	3.99*10^5^ ± 2.96*10^4^	A a
	EUC-600	1	4.15*10^5^ ± 3.11*10^4^	A
		3	4.59*10^5^ ± 3.29*10^4^	A
	GHW-350	1	4.41*10^5^ ± 1.44*10^4^	a
		3	4.68*10^5^ ± 3.26*10^4^	a
*Trichoderma* spp.	Control	0	3.81*10^3^ ± 7.01*10^2^	B b
	EUC-600	1	6.56*10^3^ ± 6.03*10^2^	AB
		3	7.25*10^3^ ± 9.45*10^2^	A
	GHW-350	1	6.53*10^3^ ± 1.02*10^3^	ab
		3	7.32*10^3^ ± 6.01*10^2^	a
Yeasts	Control	0	1.13*10^5^ ± 1.24*10^4^	A a
	EUC-600	1	1.31*10^5^ ± 1.40*10^4^	A
		3	1.25*10^5^ ± 1.80*10^4^	A
	GHW-350	1	1.33*10^5^ ± 1.58*10^4^	a
		3	1.46*10^5^ ± 1.57*10^4^	a

^#^Data in each microorganism group labeled by a different capital letter or small letter are significantly different at *P* ≤ 0.05 according to Tukey Kramer HSD test within EUC-600 and GHW-350 biochar, respectively.

**Table 2:** Relative abundance of bacteria genera related to plant growth promotion, disease suppression and other possible ecological roles that are significantly influenced by biochar amendments as identified by using the Illumina sequencing of 16S rRNA gene amplicons.

Genus	Relative abundance (%) (Mean ±SE)		*P*-value	Potential environmental role##	References$
	Control	Biochar-3%			
*Burkholderia*	9.19 ± 0.394 a#	5.97 ± 0.180 b	0.0003	PP, BCA, PGP, N_2_ fixation	83–85
*Massilia*	3.03 ± 0.480 b	4.51 ± 0.167 a	0.0272	BCA, PGP	86
*Mucilaginibacter*	6.80 ± 0.361 a	0.61 ± 0.042 b	<0.0001	PGP	87
*Rhodanobacter*	2.25 ± 0.085 b	4.03 ± 0.206 a	0.0002	BCA, Denitrification process, Aromatic compound degrader	88–90
*Sphingobium*	1.98 ± 0.074 b	2.38 ± 0.015 a	0.0370	BCA	91
*Bradyrhizobium*	2.53 ± 0.056 a	1.64 ± 0.075 b	<0.0001	N2 fixation, BCA, PGP	92
*Devosia*	0.76 ± 0.023 b	3.37 ± 0.115 a	<0.0001	N_2_ fixation	93
*Sphingomonas*	3.06 ± 0.103 a	0.98 ± 0.079 b	<0.0001	BCA, Aromatic compound degrader, PP	94–96
*Flavobacterium*	0.01 ± 0.004 b	2.34 ± 0.153 a	<0.0001	BCA, PGP (chitinolytic bacteria)	27
*Pseudomonas*	0.17 ± 0.131 b	1.46 ± 0.276 a	0.0055	BCA, PGP (chitinolytic and cellulolytic bacteria), PP	45,46,97
*Chthoniobacter*	0.22 ± 0.015 b	0.65 ± 0.080 a	0.0020	OCD	98
*Achromobacter*	0.05 ± 0.013 b	0.82 ± 0.044 a	<0.0001	BCA, PGP	99,100
*Rhizobium*	0.28 ± 0.037 b	0.51 ± 0.031 a	0.0033	N_2_ fixation, BCA, PGP (chitinolytic bacteria)	101
*Mesorhizobium*	0.22 ± 0.042 b	0.52 ± 0.027 a	0.0009	N_2_ fixation, BCA, PGP	102
*Nitratireductor*	0.19 ± 0.018 b	0.42 ± 0.035 a	0.0012	PAH degrader (pyrene)	103
*Ochrobactrum*	0.05 ± 0.044 b	0.40 ± 0.040 a	0.0009	N_2_ fixation, BCA, Aromatic compound degrader, OCD	104,105
*Cellvibrio*	0.00 ± 0.000 b	0.38 ± 0.089 a	0.0055	N_2_ fixation, PGP (cellulolytic bacteria)	23
*Brevundimonas*	0.05 ± 0.017 b	0.31 ± 0.010 a	<0.0001	PGP	106
*Peredibacter*	0.00 ± 0.000 b	0.36 ± 0.045 a	0.0002	BCA	107
*Bacillus*	0.02 ± 0.009 b	0.28 ± 0.034 a	0.0003	BCA, PG (chitinolytic and cellulolytic bacteria), PP	108,109
*Ferritrophicum*	0.02 ± 0.005 b	0.26 ± 0.019 a	<0.0001	Fe(II)-Oxidizer	110
*Comamonas*	0.02 ± 0.006 b	0.25 ± 0.028 a	0.0002	BCA, Aromatic compound degrader	111,112
*Sphingopyxis*	0.00 ± 0.002 b	0.21 ± 0.034 a	0.0009	Oil and recalcitrant polyaromatic compounds degrader, OCD	113
*Stenotrophomonas*	0.01 ± 0.006 b	0.20 ± 0.006 a	0.0050	BCA, PGP (chitinolytic bacteria), Aromatic compound degrader	114–116
*Paenibacillus*	0.01 ± 0.005 b	0.19 ± 0.026 a	0.0004	BCA, PGP (cellulolytic and chitinolytic bacteria), PAH degrader	117,118
*Novosphingobium*	0.01 ± 0.008 b	0.19 ± 0.012 a	<0.0001	BCA, PGP, Aromatic compound degrader	119,120
*Afipia*	0.02 ± 0.006 b	0.17 ± 0.010 a	<0.0001	OCD	121
*Shinella*	0.01 ± 0.008 b	0.16 ± 0.012 a	<0.0001	N_2_ fixation, OCD	122,123
*Microvirga*	0.00 ± 0.002 b	0.17 ± 0.032 a	0.0017	N_2_ fixation	124
*Azospirillum*	0.00 ± 0.000 b	0.11 ± 0.012 a	0.0001	N_2_ fixation, BCA, PGP	125
*Cytophaga*	0.01 ± 0.010 b	0.09 ± 0.026 a	0.0318	OCD (chitinolytic bacteria)	126

^#^Values of each genus (row) labeled by a different small letter are significantly different at *P* ≤ 0.05 according to Student’s t-test.

^##^Potential environmental role of bacteria genera: BCA (biocontrol agents); PGP (plant growth promotion); OCD (organic compound degradation); PP (plant pathogen) as previously described in literature.

^$^References citied in this table (reference no. 83–126) is presented in Supplementary data reference.
