# Supplementary material for: Linking the Belowground Microbial Composition, Diversity and Activity to Soilborne Disease Suppression and Growth Promotion of Tomato Amended with Biochar
Source: Sci Rep. 2017 Mar 13;7:44382. doi: 10.1038/srep44382 (PMC5347032; doi:10.1038/srep44382)
Supplement: Supplementary Information [file srep44382-s1.pdf]

Supplementary Information

Linking the Belowground Microbial Composition, Diversity and Activity to Soilborne Disease Suppression and Growth Promotion of Tomato Amended with Biochar

Amit K. Jaiswal, Yigal Elad, Indira Paudel, Ellen R. Graber, Eddie Cytryn, Omer Frenkel

Supplementary Table S1: Salient physical-chemical characteristics of biochars[#] and potting mixture

Parameter	Biochar		Potting
	EUC-600	GHW-350	Mixture
SSA[a] (m^2 g^{-1})	133	2.7	-
pH[b]	9.7	9.9	6.6
EC (dS m^{-1})	0.77	7.31	0.1
Ash (wt %)	12.9	51.4	-
C (wt %)	76.7	40.2	-
H (wt %)	1.9	1.9	-
N (wt %)	1	1.3	-
O (wt %)	7.1	5.5	-

[a]SSA= Specific surface area, measured by N_2-BET

[b]pH and electrical conductivity (EC) were measured in 1:20 w:v (weight: volume) biochar: deionized water 24 h extract. High EC of the GHW biochars are residues of salts taken up by the pepper plants during their growth (grown on saline water)

[#]Data from Jaiswal et al.[1,2]

Supplementary Table S2: Putative identifications by gas chromatograph/mass spectrometer (GC/MS) of compounds in aqueous extracts of eucalyptus woodchips (EUC) biochars produced at 600˚C (EUC-600) and greenhouse pepper plant waste (GHW) biochars produced at 350˚C (GHW-350)[#]

Compound	Chemical Group	Retention time (min)	EUC-600	GHW-350
2-Hydroxypropanoic acid	Hydroxy acid	18.379		Y[a]
Lactic acid	Hydroxy acid	18.396	Y	Y
4-Hydroxybutanoic acid	Hydroxy acid	26.660		Y
Hydroxyacetic acid (glycolic acid)	α-Hydroxy acid	19.542	Y	Y
Hexanoic acid	*n*-Alkanoic acid	19.297		Y
Heptanoic acid	*n*-Alkanoic acid	24.102		Y
Octanoic acid	*n*-Alkanoic acid	27.598		Y
Nonanoic acid	*n*-Alkanoic acid	30.554		Y
Decanoic acid	*n*-Alkanoic acid	33.208		Y
Hexadecanoic acid	*n*-Alkanoic acid	46.035	Y	Y
Octadecanoic acid	*n*-Alkanoic acid	49.573		Y
Benzoic acid	Benzoic acid	26.920		Y
3-Methylbenzoic acid	Substituted benzoic acid	29.333		Y
2-Methylbenzoic acid	Substituted benzoic acid	30.216		Y
4-Hydroxybenzoic acid	Substituted benzoic acid	35.887		Y
3-hydroxybenzoic acid	Substituted benzoic acid	37.344		Y
Salicylic acid	Monohydroxybenzoic acid	34.432		Y
Cyperaquinone	Benzofuran	39.205		Y
Benzenepropanoic acid	Aromatic organic acid	32.120		Y
Succinic acid	Dicarboxylic acid	29.189	Y	Y
2-Methylbutanedioic acid	Dicarboxylic acid	29.516		Y
Oxalic acid	Dicarboxylic acid	31.522		Y
Glutaric acid	Dicarboxylic acid	31.831		Y
1,2-Butanediol	Diol	22.163		Y
Glycerol	Polyol	27.898		Y
Pyroglutamic acid	Amino acid derivative	34.765		Y

Methyl α-d-lyxofuranoside	Anhydrosugar	38.043		Y
1,6-Anhydroglucose	Anhydrosugar	38.986	Y	Y

[#]Data from Jaiswal et al.[2]

[a]Y= present

Supplementary Table S3: Base level microbiome of potting mixture. Bacterial composition at order level as identified by using the Illumina sequencing of 16S rRNA gene amplicons.

**Order**	**Relative abundance (%) (Mean ±SE)**
Rhizobiales	9.7 ± 0.55
Sphingobacteriales	14.4 ± 0.90
Acidobacteriales	19.1 ± 1.03
Xanthomonadales	16.8 ± 0.84
Burkholderiales	3.1 ± 0.81
Myxococcales	0.9 ± 0.06
Rhodospirillales	2.9 ± 0.15
Opitutales	0.3 ± 0.00
Caulobacterales	1.8 ± 0.05
Micrococcales	1.7 ± 0.17
Solirubrobacterales	2.8 ± 0.24
Acidimicrobiales	3.8 ± 0.32
Sphingomonadales	0.3 ± 0.08
Planctomycetales	0.7 ± 0.06
Flavobacteriales	0.9 ± 0.24
Gaiellales	1.0 ± 0.09
Frankiales	1.5 ± 0.06
Pseudomonadales	0.8 ± 0.31
Gemmatimonadales	0.1 ± 0.03
Cytophagales	0.3 ± 0.13
Nitrosomonadales	0.1 ± 0.02
Legionellales	0.5 ± 0.04

Bacillales	0.5 ± 0.14
Streptomycetales	0.4 ± 0.06
Bdellovibrionales	0.1 ± 0.01
Others	15.5 ± 0.37

Supplementary Table S4: Analysis of variance (ANOVA) to determine the effect of interaction between biochar types and concentration of biochar on disease of tomato caused by FORL

Source	Collapsed plant (%)[a]	AUMPC (%×days)[b]	Disease severity (%)[a]
ANOVA			
Model	<0.0001[*]	0.0001[*]	<0.0001[*]
R^2	0.6579	0.6270	0.6894
P-value			
Biochar type (B)	0.0137[*]	0.0218[*]	0.0105[*]
Concentration (C)	<0.0001[*]	<0.0001[*]	<0.0001[*]
B×C	0.2750	0.4393	0.6920

[a]Values were normalized by an arcsine square root transformation before analysis

[b]Area under mortality progress curve

[*]Significant at $P \leq 0.05$

Supplementary Table S5: Analysis of variance (ANOVA) to determine the effect of interaction between FORL inoculation (inoculated and non-inoculated), biochar type and biochar concentration on plant growth and physiological parameters.

Source	Canopy dry weight (g)	Fresh root weight (g)	Photosynthesis rate (μmol CO_2/m^2 sec)	Stomatal conductance (mol H_2O/m^2 sec)
ANOVA				
Model	<0.0001*	<0.0001*	<0.0001*	<0.0001*
R^2	0.9943	0.8822	0.7708	0.9551
P-value				
FORL inoculation (I)	<0.0001*	<0.0001*	<0.0001*	<0.0001*
Biochar type (B)	0.0142*	<0.0001*	0.0114*	<0.0001*
Concentration (C)	0.0089*	0.0003*	<0.0001*	<0.0001*
I×B	0.9065	0.4186	0.8776	0.0081*
I×C	0.3565	0.7524	0.0416*	0.0001*
B×C	0.8931	0.1041	0.4361	0.0095*
I×B×C	0.6128	0.8687	0.5706	0.0644

*Significant at $P < 0.05$

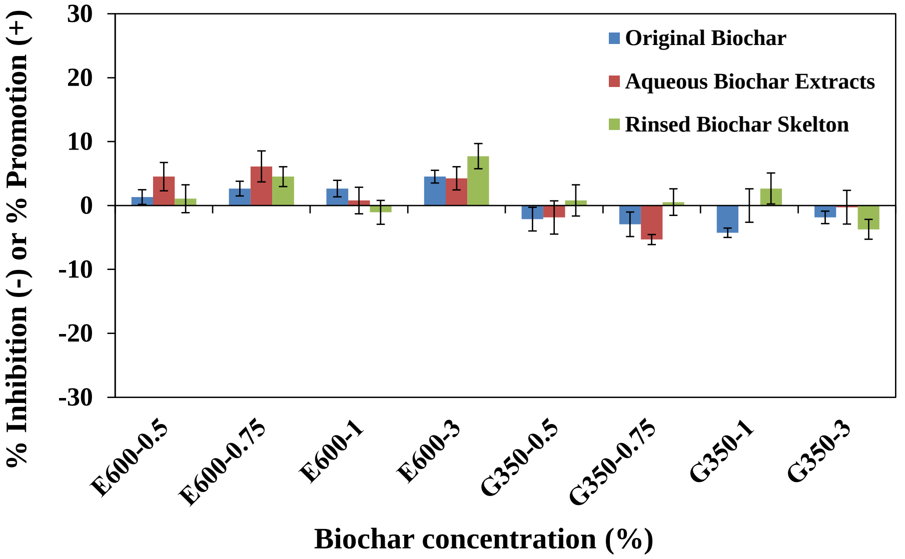

Supplementary Figure S1: *In vitro* radial mycelial growth inhibition (–) or promotion (+) for FORL exposed to increasing concentration of EUC-600 and GHW-350 biochar in 3 different form: original unwashed biochar (■), aqueous biochar extracts (■), and washed biochar (■). Bars represent the standard error.

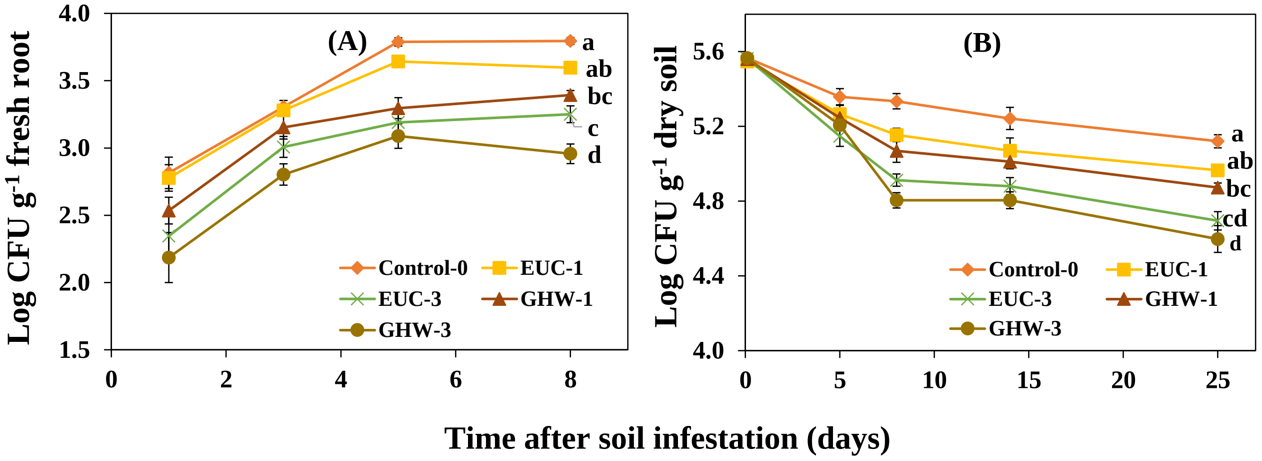

Supplementary Figure S2: Effect of eucalyptus (EUC-600) and greenhouse waste (GHW-350) biochar amendments at concentrations of 0, 1, and 3% on *Fusarium* root colonization (A), and *Fusarium* survival in potting mixture (B). The counts of *Fusarium* are presented as log CFU g^{-1} fresh root or dry potting medium. Data points labeled by a different letters are significantly different at

P≤0.05 according to Tukey Kramer HSD test within each examined dates. Bars represent the standard error.

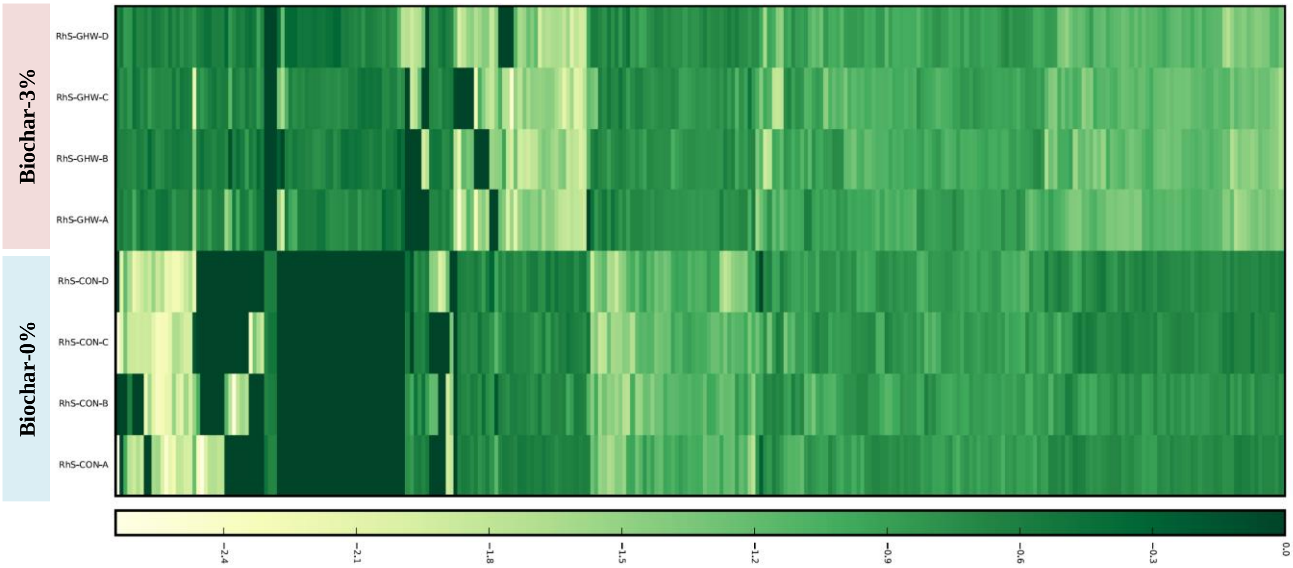

Supplementary Figure S3: Heatmap derived from most abundant OTUs (at least 50 sequences in total) of biochar amended and non-amended control rhizosphere. Each row in the heatmap represents a sample and each column in the heatmap represents an OTU (97% identity), and color intensity for each OTU is inversely proportional to abundance *i.e.* higher OTU abundance shown at minimum color intensity.

Supplementary Table S6: Effect of eucalyptus (EUC-600) and greenhouse waste (GHW-350) biochar at increasing concentration (0, 0.5, 1, and 3%) on plant growth and physiological parameters (Mean ± SE)

Inoculation	Biochar type	Dose (%)	Plant height (cm)	Leaves no. (per plant)	Transpiration (mol H_2O m^{-2} sec)	ETR (μ mol e^- m^{-2} sec)	Total chlorophyll (μg cm^{-2})	Carotenoid (μg cm^{-2})	Membrane leakage (roots-%)	Membrane leakage (hypocotyls-%)	Membrane leakage (leaves-%)
Non-Inoculated	Control	0	36.1 ±0.93 *B, b*[#]	8.3 ±0.13 *A, a*	4.4 ±0.33 B, b	77.4 ±3.22 B, b	39.3 ±1.16 A, a	6.4 ±0.24 A, a	61.1 ±4.19 a	50.0 ±2.98 a	63.5 ±2.69 a
	EUC-600	0.5	37.9 ±1.11 *AB*	8.3 ± 0.24 *A*	5.6 ±0.26 A	82.3 ±2.84 B	43.2 ± 1.19 A	7.6 ±0.21 A	ND[##]	ND	ND
		1	39.1 ±0.88 *AB*	8.4 ±0.12 *A*	5.8 ±0.28 A	85.6 ±1.58 B	43.5 ±1.65 A	6.9 ±0.35 A	ND	ND	ND
		3	41.1 ±0.94 *A*	8.9 ±0.13 *A*	6.2 ±0.25 A	95.9 ± 3.25 A	44.5 ±1.00 A	7.4 ±0.30 A	ND	ND	ND
		P-value	0.0354	0.1102	0.0029	0.0038	0.0602	0.0362	ND	ND	ND
	GHW-350	0.5	39.7 ± 1.09 *ab*	8.8 ±0.23 *a*	6.4 ±0.23 a	91.0 ±2.97 a	43.0 ±1.45 a	6.9 ±0.20 a	ND	ND	ND
		1	41.5 ±0.90 *a*	8.9 ±0.27 *a*	6.2 ±0.36 a	97.1 ±3.54 a	44.1 ±1.65 a	6.9 ±0.22 a	ND	ND	ND
		3	43.0 ±0.9 *a*	8.8 ±0.2 *a*	6.7 ±0.21 a	101.7 ±4.68 a	44.4 ±1.04 a	7.1 ±0.30 a	68.7 ±1.48 a	54.3 ±4.78 a	60.0 ±1.17 a
		P-value	0.0049	0.3075	0.0003	0.0016	0.0740	0.1990	0.1404	0.4718	0.2773
Inoculated	Control	0	24.4 ±0.81 *B, c*	6.9 ±0.19 A, b	0.6 ±0.09 C, c	30.3 ±3.41 C, c	35.0 ±0.61 C, c	6.4 ±0.20 A, b	ND	ND	ND
	EUC-600	0.5	25.4 ±1.30 *B*	7.1 ±0.23 A	1.3 ±0.16 BC	61.7 ±5.89 B	39.4 ±0.55 B	6.9 ±0.15 A	ND	ND	ND
		1	28.0 ±0.80 *AB*	7.2 ±0.27 A	2.1 ±0.25 AB	86.3 ±4.11 A	39.0 ±1.02 B	7.0 ±0.25 A	ND	ND	ND
		3	30.7 ±0.73 *A*	7.6 ±0.24 A	2.9 ±0.33 A	89.2 ±4.25 A	42.7 ±0.96 A	6.9 ±0.29 A	ND	ND	ND
		P-value	0.0010	0.2447	<0.0001	<0.0001	0.0002	0.2778	ND	ND	ND
	GHW-350	0.5	28.3 ±0.92 *b*	7.9 ±0.24 a	1.2 ±0.11 b	74.4 ±4.59 b	43.3 ±0.87 b	6.5 ±0.29 b	ND	ND	ND
		1	32.0 ±1.03 *ab*	7.8 ±0.18 a	3.1 ±0.20 a	95.0 ±3.39 a	47.6 ±1.02 a	6.8 ±0.18 ab	ND	ND	ND
		3	31.4 ±0.88 *a*	7.9 ±0.19 a	4.6 ±0.28 a	97.4 ±1.62 a	48.6 ±0.97 a	7.6 ±0.21 a	ND	ND	ND
		P-value	<0.0001	0.0065	<0.0001	<0.0001	<0.0001	0.0152	ND	ND	ND

[#] Mean ± SE, Values labeled by a different capital letter and small letter are significantly different at $P \leq 0.05$ according to Tukey Kramer HSD test within EUC-600 and GHW-350 biochar, respectively; [##]ND= not determined; ETR= Electron transport rate

Supplementary Table S7: Effect of eucalyptus (EUC-600) and greenhouse waste (GHW-350) biochar on tomato leaves nutrient and potting mixture pH and water status (Mean ± SE)

Biochar type	Dose (%)	Tomato leaves							Potting mix	
		N (g/kg)	P (g/kg)	K (g/kg)	Ca (g/kg)	Mg (g/kg)	Mn (mg/kg)	Zn (mg/kg)	Moisture content (%)	pH
Control	0	47.9 ± 1.32 a[#]	8.5 ± 0.06 a	32.7 ± 0.90 a	29.6 ± 2.35 a	3.2 ± 0.11 a	201.0 ± 18.20 a	34.2 ± 2.45 a	50.4 ± 1.78 a	6.5 ± 0.04 d
EUC-600	1	ND[##]	ND	ND	ND	ND	ND	ND	51.2 ± 1.61 a	6.8 ± 0.02 c
	3	49.2 ± 2.00 a	8.7 ± 0.42 a	33.6 ± 1.54 a	28.1 ± 2.18 a	3.2 ± 0.32 a	198.3 ± 17.10 a	35.8 ± 2.20 a	49.4 ± 0.56 a	6.9 ± 0.01 b
GHW-350	1	ND	ND	ND	ND	ND	ND	ND	48.7 ± 2.93 a	7.0 ± 0.04 b
	3	49.2 ± 1.13 a	8.9 ± 0.32 a	35.6 ± 1.86 a	30.0 ± 1.15 a	3.4 ± 0.25 a	205.0 ± 7.64 a	35.0 ± 2.89 a	48.9 ± 1.38 a	7.3 ± 0.01 a

[#] Mean ± SE, Values labeled by a different small letter are significantly different at $P \leq 0.05$ according to Tukey Kramer HSD; [##]ND= not determined

Supplementary Table S8: Effect of greenhouse waste (GHW-350) biochar on relative abundances of rhizosphere bacterial composition at order level as identified by using the Illumina sequencing of 16S rRNA gene amplicons.

**Order**	**Relative abundance (%) (Mean ±SE)**		***P*-value**
	**Control**	**Biochar-3%**	
Burkholderiales	14.3 ± 0.89 b[#]	18.0 ± 0.27 a	0.0073
Rhizobiales	17.7 ± 0.56 a	18.4 ± 0.23 a	0.2547
Sphingobacteriales	11.1 ± 0.57 a	12.3 ± 0.18 a	0.0873
Acidobacteriales	20.1 ± 0.39 a	6.5 ± 0.15 b	<0.0001
Xanthomonadales	5.0 ± 0.15 b	7.3 ± 0.31 a	0.0005
Sphingomonadales	5.4 ± 0.45 a	4.4 ± 0.23 a	0.1037
Flavobacteriales	0.2 ± 0.05 b	2.9 ± 0.16 a	<0.0001
Caulobacterales	1.3 ± 0.14 b	2.6 ± 0.08 a	0.0002
Pseudomonadales	0.2 ± 0.13 b	1.9 ± 0.61 a	0.0317
Rhodospirillales	3.1 ± 0.15 a	2.4 ± 0.05 b	0.0044
Actinomycetales	2.1 ± 0.09 s	2.4 ± 0.45 a	0.5523
Cytophagales	0.2 ± 0.03 b	1.9 ± 0.17 a	<0.0001
Myxococcales	1.0 ± 0.03 b	1.4 ± 0.09 a	0.0037
Frankiales	2.0 ± 0.20 a	1.3 ± 0.07 b	0.0294
Methylophilales	0.0 ± 0.00 b	0.1 ± 0.02 a	0.0001
Planctomycetales	1.0 ± 0.03 a	1.0 ± 0.04 a	0.5513
Verrucomicrobiales	0.0 ± 0.01 b	0.8 ± 0.13 a	0.0012
Bdellovibrionales	0.3 ± 0.02 b	0.7 ± 0.06 a	0.0003
Gemmatimonadales	0.5 ± 0.04 a	0.6 ± 0.08 a	0.3986
Nitrosomonadales	0.1 ± 0.02 b	0.7 ± 0.05 a	<0.0001
Bacillales	0.1 ± 0.02 b	0.6 ± 0.07 a	0.0002
Rhodocyclales	0.0 ± 0.00 b	0.6 ± 0.04 a	<0.0001
Opitutales	0.1 ± 0.02 b	0.3 ± 0.07 a	0.0167
Enterobacteriales	0.1 ± 0.07 a	0.0 ± 0.01 a	0.2055
Chlamydiales	0.0 ± 0.02 b	0.3 ± 0.11 a	0.0366
Others	14.2 ± 0.20 a	10.5 ± 0.40 b	0.0002

[#]Values of each bacterial order (row) labeled by a different small letter are significantly different at

P≤0.05 according to Student’s t-test.

References

1 Jaiswal, A. K., Elad, Y., Graber, E. R. & Frenkel, O. *Rhizoctonia solani* suppression and plant growth promotion in cucumber as affected by biochar pyrolysis temperature, feedstock and concentration. *Soil Biol. Biochem.* **69**, 110-118; DOI:10.1016/j.soilbio.2013.10.051 (2014).

2 Jaiswal, A. K., Frenkel, O., Elad, Y., Lew, B. & Graber, E. R. Non-monotonic influence of biochar dose on bean seedling growth and susceptibility to *Rhizoctonia solani*: the “Shifted R_{max}-Effect”. *Plant Soil,* **395**, 125–140; DOI:10.1007/s11104-014-2331-2 (2015).

References (no. 83-126) mentioned below here is of the Table 2 presented in main manuscript

83 Jacobs, J. L. *et al.* Identification and onion pathogenicity of *Burkholderia cepacia* complex isolates from the onion rhizosphere and onion field soil. *Appl. Environ. Microbiol.* **74**, 3121-3129; DOI:10.1128/aem.01941-07 (2008).

84 Fox, A., Kwapinski, W., Griffiths, B. S. & Schmalenberger, A. The role of sulfur- and phosphorus-mobilizing bacteria in biochar-induced growth promotion of *Lolium perenne*. *FEMS Microbiol. Ecol.* **90**, 78-91; DOI:10.1111/1574-6941.12374 (2014).

85 Paungfoo-Lonhienne, C. *et al.* Crosstalk between sugarcane and a plant-growth promoting *Burkholderia* species. *Sci. Rep.* **6**; DOI:10.1038/srep37389 (2016).

86 Ofek, M., Hadar, Y. & Minz, D. Ecology of root colonizing *Massilia* (*Oxalobacteraceae*). *Plos One* **7**; DOI:10.1371/journal.pone.0040117 (2012).

87 Madhaiyan, M. *et al.* Mucilaginibacter gossypii sp. nov. and Mucilaginibacter gossypiicola sp. nov., plant-growth-promoting bacteria isolated from cotton rhizosphere soils. *Int. J. Syst. Evol. Microbiol.* **60**, 2451-2457; DOI:10.1099/ijs.0.018713-0 (2010).

88 De Clercq, D. *et al. Rhodanobacter spathiphylli* sp nov., a gammaproteobacterium isolated from the roots of *Spathiphyllum* plants grown in a compost-amended potting mix. *Int. J. Syst. Evol. Microbiol.* **56**, 1755-1759; DOI:10.1099/ijs.0.64131-0 (2006).

89 Green, S. J. *et al.* Denitrifying bacteria from the genus *Rhodanobacter* dominate bacterial communities in the highly contaminated subsurface of a nuclear legacy waste site. *Appl. Environ. Microbiol.* **78**, 1039-1047; DOI:10.1128/aem.06435-11 (2012).

90 Kanaly, R. A., Harayama, S. & Watanabe, K. *Rhodanobacter* sp. strain BPC1 in a benzo a pyrene-mineralizing bacterial consortium. *Appl. Environ. Microbiol.* **68**, 5826-5833; DOI:10.1128/aem.68.12.5826-5833 (2002).

91 Bruggen, A. H. C. v., Francis, I. M. & Jochimsen, K. N. Non-pathogenic rhizosphere bacteria belonging to the genera Rhizorhapis and Sphingobium provide specific control of lettuce corky root disease caused by species of the same bacterial genera. *Plant Pathol.* **63**, 1384-1394; DOI:10.1111/ppa.12212 (2014).

92 Omar, S. A. & Abd-Alla, M. H. Biocontrol of fungal root rot diseases of crop plants by the use of *Rhizobia* and *Bradyrhizobia*. *Folia Microbiol.* **43**, 431-437; DOI:10.1007/bf02818587 (1998).

93 Rivas, R. *et al.* A new species of Devosia that forms a unique nitrogen-fixing root-nodule symbiosis with the aquatic legume Neptunia natans (L.f.) Druce. *Appl. Environ. Microbiol.* **68**, 5217-5222; DOI:10.1128/aem.68.11.5217-5222.2002 (2002).

94 Innerebner, G., Knief, C. & Vorholt, J. A. Protection of *Arabidopsis thaliana* against Leaf-Pathogenic *Pseudomonas syringae* by *Sphingomonas* Strains in a Controlled Model System. *Appl. Environ. Microbiol.* **77**, 3202-3210; DOI:10.1128/aem.00133-11 (2011).

95 Fredrickson, J. K. *et al.* Aromatic-degrading Sphingomonas isolates from the deep subsurface. *Appl. Environ. Microbiol.* **61**, 1917-1922 (1995).

96 Buonaurio, R. *et al. Sphingomonas melonis* sp nov., a novel pathogen that causes brown spots on yellow Spanish melon fruits. *Int. J. Syst. Evol. Microbiol.* **52**, 2081-2087; DOI:10.1099/ijs.0.02063-0 (2002).

97 Hirano, S. S. & Upper, C. D. Population biology and epidemiology of Pseudomonas syringae. *Annu. Rev. Phytopathol.* **28**, 155-177; DOI:10.1146/annurev.py.28.090190.001103 (1990).

98 Kant, R. *et al.* Genome sequence of *Chthoniobacter flavus* Ellin428, an aerobic heterotrophic soil bacterium. *J. Bacteriol.* **193**, 2902-2903; DOI:10.1128/jb.00295-11 (2011).

99 Ma, Y., Rajkumar, M. & Freitas, H. Inoculation of plant growth promoting bacterium *Achromobacter xylosoxidans* strain Ax10 for the improvement of copper phytoextraction by *Brassica juncea*. *J. Environ. Manage.* **90**, 831-837; DOI:10.1016/j.jenvman.2008.01.014 (2009).

100 Moretti, M., Gilardi, G., Gullino, M. & Garibaldi, A. Biological control potential of *Achromobacter xylosoxydans* for suppressing Fusarium wilt of tomato. *Int. J. Bot* **4**, 369-375 (2008).

101 Kumar, H., Dubey, R. C. & Maheshwari, D. K. Effect of plant growth promoting rhizobia on seed germination, growth promotion and suppression of Fusarium wilt of fenugreek (*Trigonella foenum*-graecum L.). *Crop Protect.* **30**, 1396-1403; DOI:10.1016/j.cropro.2011.05.001 (2011).

102 Laranjo, M., Alexandre, A. & Oliveira, S. Legume growth-promoting rhizobia: An overview on the *Mesorhizobium* genus. *Microbiol. Res.* **169**, 2-17; DOI:10.1016/j.micres.2013.09.012 (2014).

103 Lai, Q. L. *et al. Nitratireductor pacificus* sp nov., isolated from a pyrene-degrading consortium. *Int. J. Syst. Evol. Microbiol.* **61**, 1386-1391; DOI:10.1099/ijs.0.024356-0 (2011).

104 Arulazhagan, P. & Vasudevan, N. Biodegradation of polycyclic aromatic hydrocarbons by a halotolerant bacterial strain *Ochrobactrum* sp. VA1. *Mar. Pollut. Bull.* **62**, 388-394; DOI:10.1016/j.marpolbul.2010.09.020 (2011).

105 Trujillo, M. E. *et al.* Nodulation of Lupinus albus by strains of Ochrobactrum lupini sp nov. *Appl. Environ. Microbiol.* **71**, 1318-1327; DOI:10.1128/aem.71.3.1318-1327.2005 (2005).

106 Singh, N. *et al.* Brevundimonas diminuta mediated alleviation of arsenic toxicity and plant growth promotion in Oryza sativa L. *Ecotoxicol. Environ. Saf.* **125**, 25-34; DOI:10.1016/j.ecoenv.2015.11.020 (2016).

107 Manczinger, L. *et al.* (Google Patents, 2013).

108 Yuan, Y. H. & Gao, M. Y. Genomic analysis of a ginger pathogen *Bacillus pumilus* providing the understanding to the pathogenesis and the novel control strategy. *Sci. Rep.* **5**; DOI:10.1038/srep10259 (2015).

109 Kloepper, J. W., Ryu, C. M. & Zhang, S. A. Induced systemic resistance and promotion of plant growth by *Bacillus* spp. *Phytopathology* **94**, 1259-1266; DOI:10.1094/phyto.2004.94.11.1259 (2004).

110 Weiss, J. V. *et al.* Characterization of neutrophilic Fe(II)-oxidizing bacteria isolated from the rhizosphere of wetland plants and description of Ferritrophicum radicicola gen. nov sp nov., and Sideroxydans paludicola sp nov. *Geomicrobiol. J.* **24**, 559-570; DOI:10.1080/01490450701670152 (2007).

111 Bae, H. S., Lee, J. M., Kim, Y. B. & Lee, S. T. Biodegradation of the mixtures of 4-chlorophenol and phenol by *Comamonas testosteroni* CPW301. *Biodegradation* **7**, 463-469; DOI:10.1007/bf00115293 (1997).

112 El-Banna, N. M. Antifungal activity of *Comamonas acidovorans* isolated from water pond in south Jordan. *Afr. J. Biotechnol.* **6**, 2216-2219 (2007).

113 Garcia-Romero, I. *et al.* Genomic analysis of the nitrate-respiring *Sphingopyxis granuli* (formerly *Sphingomonas macrogoltabida*) strain TFA. *BMC Genomics* **17**; DOI:10.1186/s12864-016-2411-1 (2016).

114 Juhasz, A. L., Stanley, G. A. & Britz, M. L. Microbial degradation and detoxification of high molecular weight polycyclic aromatic hydrocarbons by *Stenotrophomonas maltophilia* strain VUN 10,003. *Lett. Appl. Microbiol.* **30**, 396-401; DOI:10.1046/j.1472-765x.2000.00733.x (2000).

115 Berg, G., Egamberdieva, D., Lugtenberg, B. & Hagemann, M. in *Symbioses and Stress: Joint Ventures in Biology* (eds Joseph Seckbach & Martin Grube) 445-460 (Springer Netherlands, 2010).

116 Suckstorff, I. & Berg, G. Evidence for dose-dependent effects on plant growth by Stenotrophomonas strains from different origins. *J. Appl. Microbiol.* **95**, 656-663; DOI:10.1046/j.1365-2672.2003.02021.x (2003).

117 Daane, L. L. *et al.* PAH-degradation by *Paenibacillus* spp. and description of *Paenibacillus naphthalenovorans* sp nov., a naphthalene-degrading bacterium from the rhizosphere of salt marsh plants. *Int. J. Syst. Evol. Microbiol.* **52**, 131-139; DOI: 10.1099/00207713-52-1-131 (2002).

118 Timmusk, S. & Wagner, E. G. H. The plant-growth-promoting rhizobacterium Paenibacillus polymyxa induces changes in Arabidopsis thaliana gene expression: a possible connection between biotic and abiotic stress responses. *Mol. Plant-Microbe Interact.* **12**, 951-959; DOI:10.1094/MPMI.1999.12.11.951 (1999).

119 Sohn, J. H., Kwon, K. K., Kang, J.-H., Jung, H.-B. & Kim, S.-J. Novosphingobium pentaromativorans sp. nov., a high-molecular-mass polycyclic aromatic hydrocarbon-degrading bacterium isolated from estuarine sediment. *Int. J. Syst. Evol. Microbiol.* **54**, 1483-1487; DOI:10.1099/ijs.0.02945-0 (2004).

120 Sang, M. K. *et al.* Biocontrol of Phytophthora blight and anthracnose in pepper by sequentially selected antagonistic *Rhizobacteria* against *Phytophthora capsici*. *Plant Pathol. J.* **29**, 154-167; DOI:10.5423/PPJ.OA.07.2012.0104 (2013).

121 Moosvi, S. A. *et al.* Isolation and properties of methanesulfonate-degrading Afipia felis from Antarctica and comparison with other strains of A. felis. *Environ. Microbiol.* **7**, 22-33; DOI:10.1111/j.1462-2920.2004.00661.x (2005).

122 Wu, H. B. *et al.* Biodegradation mechanism of 1H-1,2,4-triazole by a newly isolated strain *Shinella* sp. NJUST26. *Sci. Rep.* **6**; DOI:10.1038/srep29675 (2016).

123 Lin, D. X. *et al.* Shinella kummerowiae sp. nov., a symbiotic bacterium isolated from root nodules of the herbal legume Kummerowia stipulacea. *Int. J. Syst. Evol. Microbiol.* **58**, 1409-1413; DOI:10.1099/ijs.0.65723-0 (2008).

124 Ardley, J. K. *et al. Microvirga lupini* sp. nov., *Microvirga lotononidis* sp. nov. and *Microvirga zambiensis* sp. nov. are alphaproteobacterial root-nodule bacteria that specifically nodulate and fix nitrogen with geographically and taxonomically separate legume hosts. *Int. J. Syst. Evol. Microbiol.* **62**, 2579-2588; DOI:10.1099/ijs.0.035097-0 (2012).

125 Tortora, M. L., Díaz-Ricci, J. C. & Pedraza, R. O. Azospirillum brasilense siderophores with antifungal activity against Colletotrichum acutatum. *Arch. Microbiol.* **193**, 275-286; DOI:10.1007/s00203-010-0672-7 (2011).

126 Cottrell, M. T. & Kirchman, D. L. Natural assemblages of marine proteobacteria and members of the *Cytophaga-Flavobacter* cluster consuming low- and high-molecular-weight dissolved organic matter. *Appl. Environ. Microbiol.* **66**, 1692-1697; DOI:10.1128/aem.66.4.1692-1697 (2000).
